# Sexual dimorphism in phenotypic plasticity and persistence under environmental change: An extension of theory and meta‐analysis of current data

**DOI:** 10.1111/ele.14005

**Published:** 2022-03-25

**Authors:** Sandra Hangartner, Carla M. Sgrò, Tim Connallon, Isobel Booksmythe

**Affiliations:** ^1^ School of Biological Sciences Monash University Clayton Victoria Australia

**Keywords:** climate change, local adaptation, population persistence, selection, sex differences

## Abstract

Populations must adapt to environmental changes to remain viable. Both evolution and phenotypic plasticity contribute to adaptation, with plasticity possibly being more important for coping with rapid change. Adaptation is complex in species with separate sexes, as the sexes can differ in the strength or direction of natural selection, the genetic basis of trait variation, and phenotypic plasticity. Many species show sex differences in plasticity, yet how these differences influence extinction susceptibility remains unclear. We first extend theoretical models of population persistence in changing environments and show that persistence is affected by sexual dimorphism for phenotypic plasticity, trait genetic architecture, and sex‐specific selection. Our models predict that female‐biased adaptive plasticity—particularly in traits with modest‐to‐low cross‐sex genetic correlations—typically promotes persistence, though we also identify conditions where sexually monomorphic or male‐biased plasticity promotes persistence. We then perform a meta‐analysis of sex‐specific plasticity under manipulated thermal conditions. Although examples of sexually dimorphic plasticity are widely observed, systematic sex differences are rare. An exception—cold resistance—is systematically female‐biased and represents a trait wherein sexually dimorphic plasticity might elevate population viability in changing environments. We discuss our results in light of debates about the roles of evolution and plasticity in extinction susceptibility.

## Graphical abstract

We extend theoretical models of population persistence in changing environments and show that female‐biased adaptive plasticity typically promotes population persistence. We then performed a meta‐analysis to systematically test for sex differences in plasticity under manipulated thermal conditions. Despite individual examples of sexually dimorphic plasticity, systematic differences were rare (the only exception being female‐biased plasticity for cold resistance).

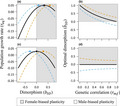

## INTRODUCTION

Mean temperature and temperature extremes are expected to increase globally over the coming decades, posing a major threat to the earth's biodiversity (Collins et al., 2013). Populations that cannot migrate to favourable habitats, or tolerate changing thermal conditions via plastic or evolved responses, may ultimately decline and go extinct (Williams et al., 2008). Extinctions linked to global environmental change have been widely documented (Parmesan & Yohe, 2003; Román‐Palacios & Wiens, 2020) and are expected to increase in the coming decades (Urban, 2015; Warren et al., 2018). The challenge for evolutionary biologists is to determine how evolutionary, genetic, developmental and ecological factors interact in shaping extinction susceptibilities.

In species with separate sexes, rates of adaptive evolution depend on three key population parameters: (1) the additive genetic variation in traits affecting female and male fitness, (2) the direction and strength of selection on these traits within each sex and (3) cross‐sex genetic correlations, which quantify overlap in the genetic basis of female and male trait variation (Lande, 1980; Poissant et al., 2010). Additive genetic variation for sexual dimorphism—a function of both within‐sex additive genetic variance (*V_A_*) and between‐sex genetic correlations (*r_mf_*)—determines the evolutionary capacity for phenotypic divergence between the sexes (i.e. sexual dimorphism; Matthews et al., 2019). Most traits exhibit strong and positive values for *r_mf_* (though *r_mf_* magnitudes vary among traits; see Poissant et al., 2010, Hangartner et al., 2020), which can constrain adaptation when the direction of selection differs between the sexes (i.e. selection is sexually antagonistic) and promote adaptation when directional selection is largely aligned (i.e. sexually concordant; see Lande, 1980, Bonduriansky & Chenoweth, 2009, Berger et al., 2014; Connallon, 2015, Connallon & Hall, 2016; Cally et al., 2019).

In addition to adaptive evolution, sex differences in phenotypic plasticity in response to thermal changes may also play significant roles in adaptation and population persistence during climate change (Fox et al., 2019; Garcia‐Roa et al., 2020; Stillwell et al., 2010). Phenotypic plasticity—the ability of an organism to express different phenotypes in different environments (Agrawal, 2001)—allows populations to respond quickly to new thermal conditions and is thought to be an important determinant of adaptation and persistence under rapid climate change (Chevin & Lande, 2010; Chevin et al., 2010; Hoffmann & Sgrò, 2011; Merilä & Hendry, 2014). Evolutionary theory usually models phenotypic plasticity as adaptive (but see Chevin & Hoffmann, 2017). Although we know surprisingly little about the fitness consequences of phenotypic responses to thermal variation, it has become clear that plasticity can also be maladaptive (e.g. Ghalambor et al., 2007; Huey et al., 2003), complicating predictions about the consequences of plasticity for adaptation and population persistence under environmental change (Chevin & Hoffmann, 2017). In addition, plasticity might not only differ between the sexes and/or environments in mean, but also in variance (e.g. Garcia‐Roa et al., 2020), which has important implications for population persistence and adaptation. Stressful or novel environments can, for instance, increase phenotypic variance (e.g. Ghalambor et al., 2007; Hoffmann & Merlia, 1999; Hoffmann & Parsons, 1997), which may be a source of novel adaptive variation (reviewed in Badyaev 2005). Sex differences in variance in phenotypic plasticity may be caused by sex‐specific selection (Martinossi‐Allibert et al., 2017).

Cases of sexually dimorphic plasticity have been documented in a range of animal species (e.g. Stillwell et al., 2010; see below), raising questions about possible consequences of sex‐specific plasticity for adaptation and persistence in the myriad species that are comprised of distinct sexes. For example, dimorphic plasticity might facilitate rapid adaptive responses to climate change within the more plastic sex, while simultaneously dampening the contribution of that sex to the adaptive evolutionary response of the population (e.g. Ghalambor et al., 2007), thereby placing a greater burden of selection on the less plastic sex (e.g. Connallon & Hall, 2016; Whitlock & Agrawal, 2009). These consequences of sexually dimorphic plasticity may have carry‐over effects on population dynamics, potentially influencing population growth or decline within altered habitats.

The roles of phenotypic plasticity and evolutionary adaptation in population persistence have received considerable attention in climate adaptation research (Chevin et al., 2010; Hoffmann & Sgrò, 2011; Merilä & Hendry, 2014; Merilä & Hoffmann, 2016), but the prevalence of sexually dimorphic plasticity, and its consequences for adaptation and persistence, have so far received little consideration. From a theoretical perspective, it remains unclear whether and how much sex differences in plasticity might facilitate or impede population persistence, particularly in species where population dynamics are more strongly coupled to female than to male fitness components (e.g. Riesch et al., 2016). In most species, females’ higher energetic investment in offspring production should cause population growth to be more sensitive to female than to male survival and fecundity (a scenario known as ‘female demographic dominance’; Crowley, 2000, Harts et al., 2014). Conditions of sex‐specific plasticity that maintain female fitness components in changing thermal environments could, therefore, promote population persistence, although the scenario has yet to be formally modelled.

From an empirical standpoint, our understanding of broad‐scale patterns of sex‐specific plasticity among populations, traits and environmental variables is limited. To date, sexually dimorphic plasticity in body size has received the most attention, and appears to be common, at least among insects (Stillwell et al., 2010; Teder & Tammaru, 2005). Several case studies have documented sexually dimorphic plasticity for traits linked to climatic adaptation, including cold recovery time (Ransberry et al., 2011), heat knockdown time (Cooper et al., 2012) and survival and longevity under heat stress (Cui et al., 2008; Sørensen et al., 2007). A recent meta‐analysis of 37 studies focusing on thermal acclimation of ectothermic species found no consistent pattern of sex‐specific phenotypic plasticity (Pottier et al., 2021). Despite these observations, it remains unclear whether one sex tends to be more plastic or more variable in the plastic response than the other, and which types of traits exhibit the most (or least) dimorphism in plasticity. As we illustrate further below, answers to both questions can have important implications for the dynamics of adaptation and population viability.

Here, we first extend evolutionary quantitative genetic models of population persistence in changing environments by considering the effects of sexual dimorphism in genetic variation, natural selection, and phenotypic plasticity on adaptation and population growth under directional and cyclic environmental change. Our models show that sex differences in plasticity can affect extinction susceptibility by mediating the *effective* rates of change of female and male trait optima under environmental change. We outline how the effects of sexually dimorphic plasticity on persistence are contingent on population‐specific attributes of selection, genetic variances and genetic correlations between the sexes and the dependency of population dynamics on female versus male fitness components. We then present a comprehensive literature search and meta‐analysis of studies estimating sex‐specific plasticity in response to altered thermal conditions. We use the meta‐analysis and theoretical results to evaluate whether patterns of sex‐specific plasticity are likely to hinder or enhance adaptation and persistence under sustained thermal change.

## METHODS

### Theoretical models of population persistence in changing environments

Our models build upon those of Chevin et al. (2010), who considered the dynamics of a key ecological trait mediating population growth in a changing environment, and Connallon and Hall (2016), who considered the effects of cross‐sex genetic correlations and sex differences in selection on female and male adaptation. Our extension of these models allows for sex differences in additive genetic variation, phenotypic plasticity and the strength or direction of selection on the key ecological trait. Following previous work (see Chevin et al., 2010; Connallon & Hall, 2016; Lande, 1980; Lande & Shannon, 1996), our models rely on three simplifying assumptions for tractability. First, that fitness is a Gaussian function of trait expression, with trait optima changing with the environment, and the strength of stabilising selection remaining constant. Second, plasticity in the trait is determined by a fixed reaction norm within each sex, where the reaction norm is the pattern of phenotypic expression of a single genotype across differing environmental conditions (Scheiner, 1993). Thus, although our models focus on the effects of existing sexually dimorphic plasticity on population dynamics, they do not address the reasons why sexually dimorphic plasticity might have evolved in the first place. We later return to this point by discussing environmental and genetic factors that can affect the evolution of reaction norms (Lande, 2014), particularly in the context of sex differences. Finally, we assume that genetic variances and cross‐sex genetic covariances are stable over time. This widely used approximation is most reasonable when genetic variation is attributable to many, loosely‐linked loci with small individual phenotypic effects (Bulmer, 1980; Chevin, 2013; Turelli, 1988). Empirical studies suggest that genetic variances and cross sex covariances may often remain constant across populations (e.g. Hangartner et al., 2020). For our purposes, the assumption is logistical (see Servedio et al., 2014) and not central to the primary question our models address.

We track the dynamics of sex‐specific adaptation and the intrinsic growth rate of the population—both of which are functions of the trait's expression in each sex—in an environment that changes either linearly (directionally) or cyclically over time. We focus on population dynamics at two extremes along a spectrum of female‐biased contributions to population growth: (1) *female demographic dominance*, wherein the intrinsic growth rate of the population is a function of female, but not male, adaptation to the environment; and (2) *demographic co*‐*dominance*, in which the intrinsic growth rate is equally sensitive to female and male adaptation. Female demographic dominance applies to species where males provide no parental investment aside from genes, whereas demographic co‐dominance applies to species with equal parental investment from each sex (e.g. De Lisle, 2021).

Full details of the models are presented in the Supplementary material, and we present a summary of their predictions in the Results. Table 1 summarises the key variables and parameters of the models.

**TABLE 1:** Summary of notation used in the models

*f*, *m*	Sex: *f* = female; *m* = male
*B_f_, B_m_*	Rate of change of female and male optima with changes in the environment
*b_f_, b_m_*	Sex‐specific reaction norms (phenotypic plasticity); phenotypic plasticity is adaptive, yet imperfect, when 1 > *b_f_*/*B_f_* > 0 and 1 > *b_m_*/*B_m_* >0
$\bar{b}$, $b_{\mathrm{SD}}$	$\bar{b}=\left(b_{f}+b_{m}\right)/2$ is the average of the female and male reaction norms; $b_{\mathrm{SD}}=b_{f}‐b_{m}$ is a measure of sexual dimorphism for the reaction norms
*V_A_*_,_ *_f_* , *V_A_*_,_ *_m_*	Sex‐specific additive genetic variance for the trait
*K_f_, K_m_*	Effective rate of change of female and male optima (*K_f_* = *B_f_* –*b_f_*; *K_m_* = *B_m_*–*b_m_*)
*α*	Variance‐weighted ratio of effective rate of change in male vs. female optima;$\alpha =K_{m}\sqrt{V_{A,f}}{\left(K_{f}\sqrt{V_{A,m}}\right)}^{‐1}$
*γ_f_*, *γ_m_*	Sex‐specific strengths of stabilising selection on the trait
*γ_b_*_,_ *_f_* , *γ_b_*_,_ *_m_*	Sex‐specific costs of plasticity (there is no cost of plasticity when *γ_b_*_,_ *_f_*, *γ_b_*_,_ *_m_* = 0)
*r_mf_*	Cross‐sex additive genetic correlation for the trait
$r_{eq.}$	Equilibrium intrinsic growth rate under directional environmental change
${\bar{r}}_{eq.}$	Steady‐state mean intrinsic growth rate under cyclic environmental change
*η*	Rate of directional change in the environment (change per generation)

### Meta‐analyses of sex‐specific phenotypic plasticity

#### Literature search and data collection

We compiled data on sex‐specific plasticity in response to thermal manipulations from a systematic literature survey covering major ecological, evolutionary and entomological journals (search terms and inclusion criteria are detailed in the Supplementary material, Appendix 1). We included studies of invertebrates that measured phenotypes of both sexes, separately, in at least two experimental thermal treatments in the laboratory, and where we were able to extract means and their associated errors and sample sizes for each treatment and sex.

Thermal treatments were categorised as hardening (short‐term pre‐exposure to a thermal treatment, minutes to hours), acclimation (long‐term pre‐exposure, days to weeks), rearing (thermal condition imposed throughout development) or acute treatments (exposure to extreme thermal conditions immediately preceding or during trait measurement). Traits were grouped into seven broad classes: measures of cold resistance, heat resistance, development time, gene expression, longevity, body size and survival, which were the most common trait classes found in the available studies (*k* = 306 studies matching initial search criteria; see Supplementary material). After excluding studies that applied treatments or measured traits falling outside of these categories, and studies for which sample sizes and/or standard deviations were not available, the final data set comprised 258 studies across 205 invertebrate species from the period 1929–2020. Many studies included multiple separate experiments, reporting the results of different thermal manipulations on different experimental populations and/or measuring several different phenotypic traits from the same manipulation. Across all trait classes our data set included 973 experiments (Appendix 1).

From each experiment, we extracted group means and associated errors and sample sizes for each sex and thermal treatment level. We calculated Hedges’ *d* (Borenstein et al. 2009), the standardised mean difference in trait value between treatments, separately for males and females. Hedges’ *d* allowed us to quantify sexual dimorphism in plasticity across trait classes. As many experiments included more than two treatment levels, we calculated *d* for each pairwise combination of treatment levels within an experiment, following Noble et al. (2018).

Considering the direction of plasticity allows assessment of whether the observed plasticity is likely to be adaptive. Our expectations for the direction of adaptive plasticity in each trait class were as follows:

- *Heat and cold resistance*: We expected heat treatments to increase heat resistance and cold treatments to increase cold resistance (Cossins & Bowler, 1987; Hoffmann et al., 2003).
- *Gene expression*: Our data set only included gene expression of heat shock proteins and antioxidant enzymes. Gene expression for these proteins and enzymes is expected to increase with increasing cold or heat stress, and this response is considered adaptive for naïve populations (see King & MacRae, 2015). We, therefore, expected higher gene expression as temperature manipulations increased or decreased relative to the study population's typical thermal environment. However, we note that increased gene expression is not necessarily adaptive in all circumstances, as populations that have adapted to a thermal stress may be expected to show reduced gene expression in response to these conditions (e.g. Sørensen et al., 2001).
- *Survival*: We expected heat treatments to increase survival under heat stress and cold treatments to increase survival under cold stress (Cossins & Bowler, 1987).
- *Size*, *longevity and development time*: Previous studies have shown that size, longevity and development time are usually reduced in warmer temperatures (Keil et al., 2015; Ohlberger, 2013), and we, therefore, expected to find the same pattern in our analysis. The direction of clinal patterns assessed under common garden conditions can be used to indirectly infer adaptive patterns in colder versus warmer environments, with repeated genetic clines implying convergent evolutionary responses to common patterns of spatially varying phenotypic selection (Endler, 1977). We note, however, that whether the usually smaller sizes and shorter longevities and development times found in warmer temperature treatments are adaptive remains controversial, and we caution against interpreting them as such. Genetic clines in invertebrate body size are inconclusive, with reports of both increasing and decreasing size from temperate to tropical environments (Blanckenhorn & Demont, 2004). Although there are few studies on latitudinal clines in development time, there are studies reporting faster (e.g. Blanckenhorn & Demont, 2004) as well as slower development time (e.g. Sgrò & Blows, 2007) in warmer latitudes. Clinal common garden studies of lifespan reveal mixed results. In North America lifespan in *D*. *melanogaster* has been shown to increase with increasing latitude (Schmidt & Paaby, 2008), whereas in Australia lifespan decreases with increasing latitude but only when populations from mid to high latitude are compared (Sgrò et al., 2013).

The calculation of Hedges’ *d* typically subtracts the value of the control group from the value of the manipulated group, so that positive *d* indicates a larger value for the manipulated group. However, our data set included many experiments that did not reflect this ‘control vs. manipulation’ framework, even when they used only two treatment levels. Additionally, studies were heterogeneous in experimental design: we considered studies that applied either cold or heat treatments, studies that used small or large temperature differences, with temperature treatments that fell within or outside of the focal populations’ typical thermal range, and studies that manipulated the thermal environment in different ways (e.g. manipulating acclimation time vs. acclimation temperature). Finally, the measured traits also varied in whether a higher or lower trait value indicates better performance (e.g. for lower thermal limits, smaller is better, while for survival duration, larger is better). We therefore analysed each of our seven trait classes separately. Within the development time, longevity, size and survival trait classes, we calculated *d* by subtracting the trait mean in the warmer treatment from the trait mean in the colder treatment, such that positive *d* reflects larger trait values (slower development, longer lifespan, larger size and longer survival) under colder temperatures. Positive *d* is thus broadly consistent with our expectations for plasticity in these traits (outlined above), although we would expect the opposite (i.e. negative *d*) from experiments imposing severe cold treatments, especially in the survival trait class—a possibility that we examined further in moderator analyses (see below). Within the cold resistance, heat resistance and gene expression trait classes, we calculated *d* by subtracting the trait mean in the colder treatment from the trait mean in the warmer treatment. Positive *d* reflects larger trait values under warmer temperatures for these trait classes. This is consistent with our predictions for adaptive plasticity in these traits, as larger trait values indicate better performance in the heat resistance traits (e.g. CTmax, time to knockdown), which are predicted to improve under the heat manipulations imposed, while smaller trait values indicate better performance in the cold resistance traits (e.g. CTmin, time to recovery from cold immobilisation), which are predicted to improve when cold manipulations are imposed. For gene expression, most studies investigated heat manipulations, under which we expect increased expression; again, differences in mean *d* due to aspects of study design were further examined in moderator analyses described below. We also included analyses of |*d*| (the absolute value of *d*), allowing us to examine the mean magnitude of plastic responses to temperature, regardless of the direction of the response. Where the magnitude of |*d*| is substantially greater than that of *d*, heterogeneity in the direction of plasticity, rather than constrained plastic responses to temperature, is likely to play an important role in determining mean *d*.

To quantify differences in variability between treatment levels and between the sexes, we calculated *lnCVR*, the natural log of the ratio of the coefficients of variation of two groups, and its associated variance (Nakagawa et al., 2015). We used *lnCVR_treatment_* to compare the variability of each pairwise combination of treatment levels, and *lnCVR_sex_* to compare the variability of males and females measured at each treatment level.

Effect sizes were excluded from analyses when the reported standard deviation for one or both group means was zero, and where the pooled sample size for both groups was <5. In analyses of *lnCVR*, we also excluded cases where mean trait expression was zero.

#### Distribution of the slopes of female and male reaction norms

Before performing the formal meta‐analyses, we visualised the distributions of the slopes of female and male reaction norms. To ensure slopes were comparable we only used studies that exposed each treatment group to a different temperature, and excluded studies that used other treatments, such as varying the duration of exposure to a temperature stress. For each sex, we first standardised the values at each treatment level by the mean trait value for that sex, as traits were measured on different scales in different studies. We then regressed the standardised values against temperature.

#### Meta‐analytic models

We implemented Bayesian meta‐analysis models in *R* (v.3.2.1; R Core Development Team, 2016) using the *MCMCglmm* package (Hadfield, 2010; see Supplementary material for MCMC sampling details). For each of the seven trait classes, we ran a meta‐regression to find the weighted mean value and 95% credible interval of Hedges’ *d*, including ‘sex’ as the only fixed predictor to determine whether males and females systematically differ in their plastic response to temperature. We included species identity, phylogenetic relatedness, study ID, and experiment within study as random effects, and allowed the estimated residual error variance in the models to vary between the sexes.

Estimates of *d* were weighted by their sampling error variance (*V*, see Supplementary material) using the ‘ginverse’ argument in MCMCglmm to include a variance‐covariance matrix, M, with the sampling error variance for each effect size along the diagonal. To account for non‐independence among effects from the same experiment, we modified M to include an off‐diagonal covariance between all effect sizes calculated as $cov\left(i,j\right)=r*\surd V_{i}\surd V_{j}$, where *r* (the correlation between effect sizes *i* and *j*) was assumed to be 0.5 between effect sizes within an experiment (see Booksmythe et al., 2017; Noble et al., 2018). To account for phylogenetic relatedness we constructed a phylogenetic correlation matrix using the Open Tree of Life (OTL; Hinchcliff et al., 2015, https://tree.opentreeoflife.org) to obtain a phylogenetic tree describing the hypothesised relationships among taxa included in our dataset. As this tree provides no information on evolutionary divergence times, we estimated branch lengths following Grafen (1989) to build the matrix (see Noble et al., 2018).

By applying the posterior distributions of parameters from these (Gaussian) models to the folded‐normal distribution (Noble et al., 2018), we obtained estimates of the mean and credible interval of |*d*| to determine the magnitude of plasticity in the measured traits, regardless of direction (following the ‘analyse‐then‐transform’ approach of Morrissey, 2016).

Following Nakagawa and Santos (2012) we calculated *I* ^2^ statistics for the meta‐regression of Hedges’ *d*. Total *I* ^2^ describes the proportion of heterogeneity among effect sizes that is not due to sampling error and can be partitioned into *I* ^2^ estimates for each variance component, plus residual variation. Heterogeneity was generally high (see Results), so we ran additional meta‐regression models, each including a second moderator variable crossed with sex to investigate how different traits measured and treatments used within each broader trait class contribute to the observed variation (see Table S1 for details of the moderator variables examined for each trait class).

Following the same approach as for our analyses of Hedges’ *d*, we used meta‐regression of *lnCVR* for each pairwise combination of treatment levels (*lnCVR_treatment_*) to ask whether traits were systematically more or less variable across temperature treatments. These models used sex as a predictor, and species identity, phylogenetic relatedness, study ID and experiment within study as random effects. Again, we extended these models to include additional moderators (Table S1) that might help to explain heterogeneity among effect size estimates.

We also analysed *lnCVR* between the sexes at each treatment level, to test whether traits measured in males are more or less variable than those measured in females, at any temperature. We included species identity, phylogenetic relatedness, study ID and experiment within study as random effects. We ran both an intercept‐only meta‐analysis of *lnCVR_sex_*, and a meta‐regression including the moderator variables detailed in Table S1.

Finally, we ran versions of all models to test for publication biases and additional methodological considerations, described in detail in the Supplementary material.

## RESULTS

### Sexual dimorphism for plasticity and population growth in changing environments

A population's extinction susceptibility under environmental change hinges upon its long‐term or ‘steady‐state’ intrinsic growth rate (Chevin et al., 2010; Lande & Shannon, 1996), represented in our models as *r_eq_* (or its mean at steady‐state, ${\bar{r}}_{\mathrm{eq}}$, under cyclic environmental change). Populations are destined for extinction when the long‐term intrinsic growth rate is consistently negative (*r_eq_* <0), but may persist when long‐term growth is positive (*r_eq_* >0).

#### Directional environmental change and female demographic dominance

When population dynamics primarily depend on female adaptation, then the steady‐state intrinsic growth rate of a population exposed to directional change in the environment is

(1) $$r_{\mathrm{eq}}=r_{\max}‐\frac{2}{\gamma_{f}}{\left(\frac{\eta K_{f}}{V_{A,f}}\right)}^{2}{\left(\frac{1‐r_{\mathrm{mf}}\alpha }{1‐r_{\mathrm{mf}}^{2}}\right)}^{2},$$

(see Supplementary Material; Table 1 provides a summary of notation), where *r*_max_ is the maximum intrinsic growth rate (i.e. that of a population in which the female trait mean matches the female trait optimum). The compound parameter $\alpha =K_{m}\sqrt{V_{A,f}}{\left(K_{f}\sqrt{V_{A,m}}\right)}^{‐1}$ quantifies the relative contributions of selection on males versus selection on females to the population's *evolutionary* response, with $K_{f}=\left(B_{f}‐b_{f}\right)$ and $K_{m}=\left(B_{m}‐b_{m}\right)$ representing the e*ffective* rates of change of female and male trait optima: the rates of change in the optimum (*B_f_* and *B_m_*) in excess of the trait's plastic response to environmental change (*b_f_* and *b_m_*).

**FIGURE 1:**
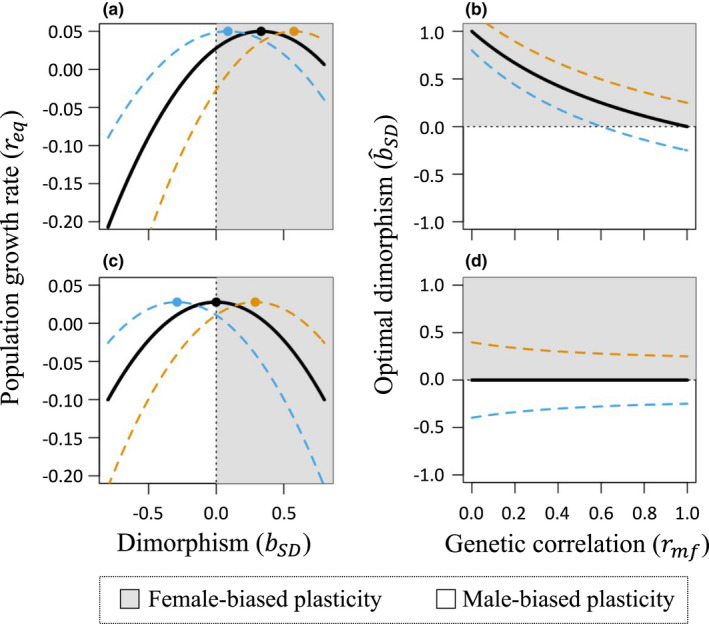
Effects of sexual dimorphism in plasticity on the steady‐state intrinsic population growth ($r_{\mathrm{eq}}$) under directional change in the environment. Under female demographic dominance (panels a, b), female‐biased plasticity (*b_SD_* >0; shaded regions) typically promotes long‐run population growth. Curves are based on Equations ((1), (2)), with parameters: *η* = 0.05; (*b_f_* + *b_m_*)/2 = 0.5, *r*_max_ = 0.05, (*V_A_*_,_ *_f_* + *V_A_*_,_ *_m_*)/2 = 0.5, (*B_f_* + *B_m_*)/2 = 1, and no cost of plasticity; *r_mf_* = 0.5 in panel (a), and *r_mf_* varies in panel (b). The black curves use *B_f_* = *B_m_*, *V_A_*_,_ *_f_* = *V_A_*_,_ *_m_*, and γ*_f_* = γ*_m_*; orange curves use *B_f_* –*B_m_* = 0.2, *V_A_*_,_ *_f_* –*V_A_*_,_ *_m_* = −0.1, and γ*_f_* = γ*_m_*; and blue curves use *B_f_* –*B_m_* = −0.2, *V_A_*_,_ *_f_* –*V_A_*_,_ *_m_* = 0.1, and γ*_f_* = γ*_m_*. Under demographic co‐dominance (i.e. where sexes contribute equally to population growth) (panels c, d), sex‐biased plasticity (*b_SD_* ≠ 0) tends to hinder population growth. Curves are based on Equation (3) in panel (c) and Equation (4) in panel (d), with the parameters to those in panels a, b. In all four panels, that shaded regions of the parameter space (*b_SD_* > 0) correspond to female‐biased plasticity, and unshaded regions *b_SD_* < 0) correspond to male‐biased plasticity

To explore the effect of sexual dimorphism for plasticity on *r_eq_*, let $b_{\mathrm{SD}}=b_{f}‐b_{m}$ represent the pattern of sexual dimorphism for plasticity. The value of $b_{\mathrm{SD}}$ that *maximises* the intrinsic growth rate is

(2) $${\hat{b}}_{\mathrm{SD}}=\frac{\frac{2}{\gamma_{f}}{\left(\frac{\eta }{V_{A,f}\left(1‐r_{\mathrm{mf}}^{2}\right)}\right)}^{2}\left(1+r_{\mathrm{mf}}\frac{\sqrt{V_{A,f}}}{\sqrt{V_{A,m}}}\right)\left(B_{f}‐\bar{b}‐r_{\mathrm{mf}}\frac{\sqrt{V_{A,f}}}{\sqrt{V_{A,m}}}\left(B_{m}‐\bar{b}\right)\right)‐\frac{\gamma_{b,f}}{2}\bar{b}}{\frac{\gamma_{b,f}}{4}+\frac{1}{\gamma_{f}}{\left(\frac{\eta }{V_{A,f}\left(1‐r_{\mathrm{mf}}^{2}\right)}\right)}^{2}{\left(1+r_{\mathrm{mf}}\frac{\sqrt{V_{A,f}}}{\sqrt{V_{A,m}}}\right)}^{2}},$$

where $\bar{b}=\left(b_{f}+b_{m}\right)/2$ is the average amount of plasticity for the trait; other terms are defined in Table 1. When costs of plasticity are negligible ($\gamma_{b,f}=0$), the cross‐sex genetic correlation is positive (*r_mf_* >0, which is typical for quantitative traits; Poissant et al., 2010; Matthews et al., 2019), and plasticity is adaptive, yet imperfect (*B_f_* > $\bar{b}$ > 0 or *B_f_* < $\bar{b}$ < 0), $r_{\mathrm{eq}}$ tends to be maximised under female‐biased plasticity (e.g. ${\hat{b}}_{\mathrm{SD}}>0$ when *B_f_* >0, as shown in Figure 1a, b). Population growth is maximised for relatively strong female biases for plasticity when the cross‐sex genetic correlation is weak ($r_{\mathrm{mf}}\ll 1$; right‐hand panel of Figure 1b), when the female optimum shifts more rapidly than the male optimum (*B_f_*/*B_m_* >1) and/or genetic variation is greater in males than females (*V_A_*_,_ *_m_*/*V_A_*_,_ *_f_* > 1; orange curves in Figure 1a, b). More modest sex biases in plasticity are favourable (with respect to *r_eq_*) when *r_mf_* is strong, male optima shift more rapidly, and genetic variation is greater in females (blue curves in Figure 1a, b). Costs of plasticity tend to reduce the optimal magnitude of the female bias for plasticity.

### Directional environmental change and demographic co‐dominance

When the population dynamics are equally affected by female and male adaptation, the long‐term intrinsic growth rate under directional change becomes

(3) $$r_{eq.}=r_{\max}‐\frac{1}{\gamma_{f}}{\left(\frac{\eta K_{f}}{V_{A,f}}\right)}^{2}{\left(\frac{1‐r_{\mathrm{mf}}\alpha }{1‐r_{\mathrm{mf}}^{2}}\right)}^{2}‐\frac{1}{\gamma_{m}}{\left(\frac{\eta K_{m}}{V_{A,m}}\right)}^{2}{\left(\frac{1‐\frac{r_{\mathrm{mf}}}{\alpha }}{1‐r_{\mathrm{mf}}^{2}}\right)}^{2},$$

(Supplementary Material; Table 1), where *r*_max_ is the growth rate in a population where female and male trait means correspond (respectively) to the female and male optima. Assuming that costs of plasticity (if they exist) are the same for each sex ($\gamma_{b,f}=\gamma_{b,m}$), the value of $b_{\mathrm{SD}}$ that maximises the intrinsic growth rate becomes

(4) $${\hat{b}}_{\mathrm{SD}}=\frac{\frac{2}{\gamma_{f}}{\left(\frac{1}{V_{A,f}}\right)}^{2}\left(1+r_{\mathrm{mf}}\frac{\sqrt{V_{A,f}}}{\sqrt{V_{A,m}}}\right)\left(B_{f}‐\bar{b}‐r_{\mathrm{mf}}\frac{\sqrt{V_{A,f}}}{\sqrt{V_{A,m}}}\left(B_{m}‐\bar{b}\right)\right)}{\frac{1}{\gamma_{m}}{\left(\frac{1}{V_{A,m}}\right)}^{2}{\left(1+r_{\mathrm{mf}}\frac{\sqrt{V_{A,m}}}{\sqrt{V_{A,f}}}\right)}^{2}+\frac{1}{\gamma_{f}}{\left(\frac{1}{V_{A,f}}\right)}^{2}{\left(1+r_{\mathrm{mf}}\frac{\sqrt{V_{A,f}}}{\sqrt{V_{A,m}}}\right)}^{2}}‐\frac{\frac{2}{\gamma_{m}}{\left(\frac{1}{V_{A,m}}\right)}^{2}\left(1+r_{\mathrm{mf}}\frac{\sqrt{V_{A,m}}}{\sqrt{V_{A,f}}}\right)\left(B_{m}‐\bar{b}‐r_{\mathrm{mf}}\frac{\sqrt{V_{A,m}}}{\sqrt{V_{A,f}}}\left(B_{f}‐\bar{b}\right)\right)}{\frac{1}{\gamma_{m}}{\left(\frac{1}{V_{A,m}}\right)}^{2}{\left(1+r_{\mathrm{mf}}\frac{\sqrt{V_{A,m}}}{\sqrt{V_{A,f}}}\right)}^{2}+\frac{1}{\gamma_{f}}{\left(\frac{1}{V_{A,f}}\right)}^{2}{\left(1+r_{\mathrm{mf}}\frac{\sqrt{V_{A,f}}}{\sqrt{V_{A,m}}}\right)}^{2}}$$

Comparison of Equations (2) and (4) suggests that long‐term population growth tends to be maximised for smaller values of $\left|b_{\mathrm{SD}}\right|$in scenarios involving demographic co‐dominance (Figure 1a, c). In the simplest case, where there is no cost of plasticity, male and female optima shift at the same rate, and stabilising selection and genetic variance are equal between the sexes ($\gamma_{b,f}=\gamma_{b,m}=0$; *B_f_* = *B_m_*; $\gamma_{f}=\gamma_{m}$; *V_A_*_,_ *_f_* = *V_A_*_,_ *_m_*), then ${\hat{b}}_{\mathrm{SD}}=0$under demographic co‐dominance (Equation (4)). In contrast, under female demographic dominance—provided plasticity is adaptive, yet imperfect ($0<\bar{b}/B_{f}<1$), and the cross‐sex genetic correlation is positive, yet imperfect (0 < *r_mf_* <1)—female‐biased plasticity promotes population growth (Equation (2) simplifies to ${\hat{b}}_{\mathrm{SD}}=2\left(B_{f}‐\bar{b}\right)\left(1‐r_{\mathrm{mf}}\right){\left(1+r_{\mathrm{mf}}\right)}^{‐1}$ when *B_f_* = *B_m_*, $\gamma_{b,f}=0$, and *V_A_*_,_ *_f_* = *V_A_*_,_ *_m_*). Sex differences in genetic variation and in the rates of change of trait optima similarly affect population growth under the two demographic scenarios (blue and orange curves in Figure 1).

#### Cyclic environmental change

Similar predictions emerge in contexts of directional and cyclic environmental change, provided the tempo of environmental cycles is slow relative to the rate of evolutionary change. With slow cycles, the relation between sexual dimorphism for plasticity (*b_SD_*) and steady‐state intrinsic population growth (${\bar{r}}_{eq.}$ denoting the mean intrinsic growth rate across a complete cycle of environmental change, at steady‐state) is the same as predicted under directional environmental change (see the Supplementary Material). In contrast, when cycles are fast relative to the tempo of evolution, so that the dynamics of sex‐specific adaptation and intrinsic growth are dominated by plastic responses to environmental change, the mean intrinsic growth rate at steady‐state under female demographic dominance is

(5) $${\bar{r}}_{eq.}=r_{\max}‐\frac{\gamma_{f}}{4}{\left(AK_{f}\right)}^{2}$$

(Supplementary Material), where *A* is the amplitude of the environmental cycle. With demographic co‐dominance, the mean steady‐state intrinsic growth rate becomes

(6) $${\bar{r}}_{eq.}=r_{\max}‐\frac{\gamma_{f}}{8}{\left(AK_{f}\right)}^{2}‐\frac{\gamma_{m}}{8}{\left(AK_{m}\right)}^{2}$$

In each case, there is no advantage to sexually dimorphic plasticity, per se. Rather, steady‐state growth is maximised (extinction susceptibility is minimised) when plasticity allows each sex to closely track its optimum. Under fast environmental cycles, sexually dimorphic plasticity is irrelevant to population growth under female demographic dominance. Sexually dimorphic plasticity can promote population growth under demographic co‐dominance when female and male optima exhibit different magnitudes of change in their optima (i.e. *B_f_* ≠ *B_m_*). In such cases, population growth increases when plasticity is adaptive and higher in the sex exhibiting greater change in its optimum (e.g. with no costs of plasticity, ${\bar{r}}_{eq.}$is maximised when *b_f_* = *B_f_* and *b_m_* = *B_m_*).

### Summary of the theoretical predictions

Our theoretical results collectively show that the consequences of sexually dimorphic plasticity for population growth depend on the nature of environmental change, the genetic basis of trait variation and the extent to which population growth depends on female versus male adaptation. Female‐biased plasticity is particularly likely to promote population growth when (*i*) species exhibit female demographic dominance, (*ii*) traits exhibit weak cross‐sex genetic correlations (0 < *r_mf_* << 1) and (*iii*) environmental change is directional or exhibits slow cycles. Under these conditions, demographic benefits of female‐biased plasticity are further enhanced by greater genetic variation in males than females (*V_A_*_,_ *_m_*/*V_A_*_,_ *_f_* > 1) and by faster rates of change in female relative to male optima (*B_f_*/*B_m_* >1). Both modifying factors disproportionately limit the evolutionary potential of females to track their optimum, whereas female‐biased plasticity compensates for these evolutionary limits and maintains high female adaptation and intrinsic population growth.

Exceptions to the conditions outlined above (*i*, *ii* and *iii*) will dampen or eliminate population benefits of female‐biased plasticity. For example, in taxa where the sexes contribute symmetrically to parental investment, and for traits with strong cross‐sex genetic correlations (0 << *r_mf_*), sex‐biased plasticity hinders population growth in directionally changing environments unless the sexes also differ for evolutionary potential (i.e. female‐biased plasticity promotes growth when *V_A_*_,_ *_m_*/*V_A_*_,_ *_f_* and *B_f_*/*B_m_* >1; male‐biased plasticity promotes growth when *V_A_*_,_ *_f_*/*V_A_*_,_ *_m_* and *B_m_*/*B_f_* >1). Under fast environmental cycles and female demographic dominance, plasticity is important for maintaining female adaptation and high population growth, yet there is no inherent benefit of sexually dimorphic plasticity. Where the sexes contribute equally to population growth, female‐biased plasticity promotes growth when the female optimum shifts more rapidly than the male optimum (*B_f_*/*B_m_* >1), whereas male‐biased plasticity is favourable when the male optimum shifts more rapidly (*B_m_*/*B_f_* >1).

### Empirical patterns of sex‐specific plasticity

**FIGURE 2:**
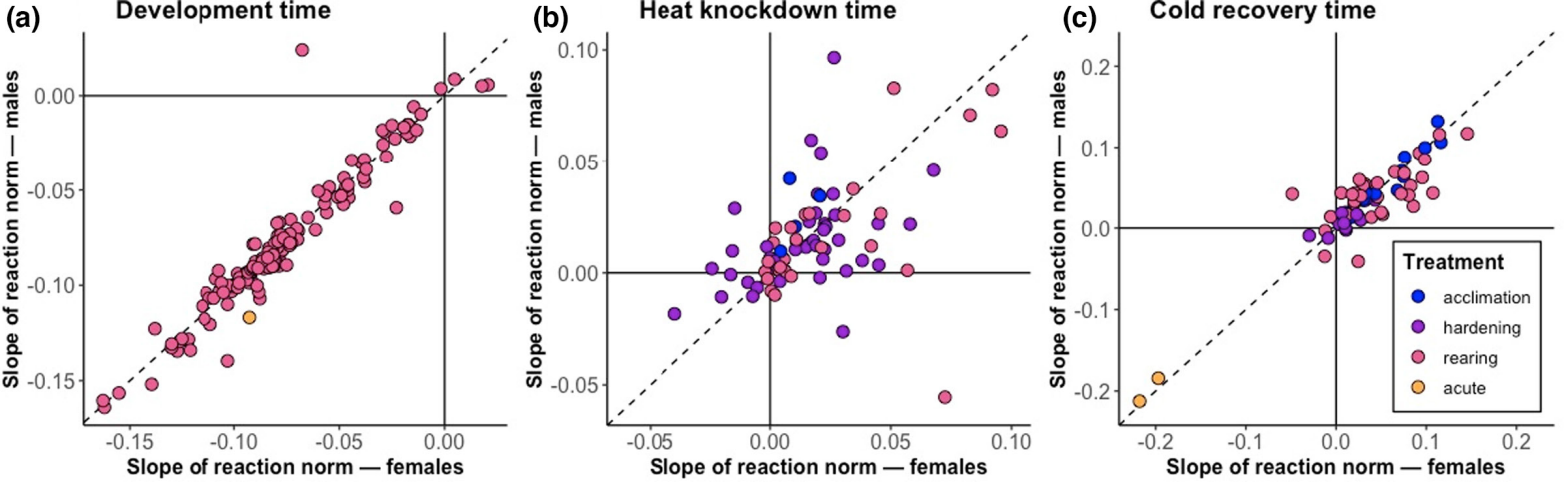
Scatter plots of female and male reaction norms (slopes of the regression of mean standardised trait values against temperature treatments) for development time, heat knockdown time and cold recovery time. Different treatment classes are represented in different colours

Empirical estimates of plasticity in all seven trait categories were positively correlated between the sexes, with most reaction norm estimates having similar magnitudes in males and females (Figure 2, S9, in which the sex‐specific distributions of mean‐standardised reaction norm estimates roughly follow the line of equality). Cold recovery time, heat knockdown time and development vary substantially in the proportion of studies showing pronounced sexual dimorphism in reaction norms (points deviating strongly from the line of equality in Figure 2). For example, reaction norm estimates that are twofold greater in one sex than the other are common among studies of heat knockdown time (Figure 2b), but rare among studies of development time (Figure 2c).

**FIGURE 3:**
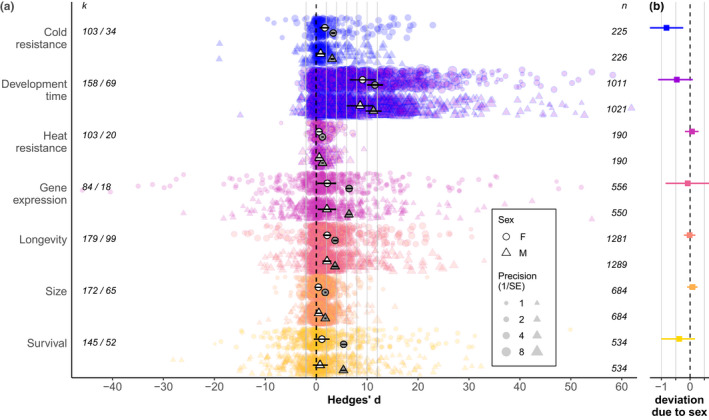
(a) Meta‐analytic mean estimates of Hedges’ *d* (open white symbols) and |*d*| (filled grey symbols) with 95% credible intervals across seven trait categories for females (circles) and males (triangles). Coloured symbols show the individual estimates of Hedges’ *d* for each contrast between treatment levels within an experiment; precision is indicated by symbol size for the individual estimates but not the mean estimates. *k* indicates the number of independent experiments/papers in each category from which *n* effect sizes were extracted for each sex. Positive *d* reflects plastic responses in the direction consistent with our main expectations (see Methods). The mean plastic responses in cold resistance, development time, heat resistance, gene expression and longevity were significant for both males and females (95% credible intervals do not contain zero). Figure S1 presents these mean estimates without the individual estimates for greater visual clarity. (b) The deviation in *d* due to sex (difference in *d* for males compared to females). Males showed significantly lower plasticity than females in cold resistance traits (95% credible interval does not contain zero); however, mean plasticity did not differ significantly between the sexes in any other trait class (see also Table 2)

The meta‐analytic mean Hedges’ *d* differed significantly from zero for the cold resistance, development time, heat resistance, gene expression and longevity trait classes, indicating broadly consistent plastic responses to temperature manipulations (Table 2, Figure 3a). We found a significant sex difference in plasticity only for cold resistance (Table 2, Figure 3b).

**TABLE 2:** Meta‐analytic mean estimates of Hedges’ *d*, and the effect of sex on *d*, across seven trait categories

	*N_species_*	*N_papers_*	*N_exp_*	*d* (95% CI)	*I* ^2^_species_ (%)	*I* ^2^_phylogeny_ (%)	*I* ^2^_paper_ (%)	*I* ^2^_experiment_ (%)	*I* ^2^_residual_ (%)	*I* ^2^_total_ (%)	*R* ^2^_conditional_	*R* ^2^_marginal_
Cold resistance Intercept Sex (male)	39	34	103	**1.76 (0.68, 2.58)** **−0.82 (−1.40, −0.24)**	0.74	1.01	4.84	14.83	77.96	99.37	0.22	0.009
Development time Intercept Sex (male)	67	69	158	9.14 (6.86, 11.78) −0.46 (−1.11, 0.09)	1.55	2.03	42.74	0.11	53.54	99.98	0.46	0.0006
Heat resistance Intercept Sex (male)	25	20	103	0.58 (0.15, 1.10) 0.07 (−0.17, 0.31)	1.92	1.79	10.57	10.79	70.73	95.79	0.26	0.002
Gene expression Intercept Sex (male)	12	18	84	**2.15 (0.30, 3.96)** −0.08 (−0.18, 0.29)	1.50	1.46	0.82	13.60	82.17	99.56	0.17	0.0005
Longevity Intercept Sex (male)	92	99	179	**2.15 (1.35, 2.99)** −0.02 (−0.21, 0.18)	3.66	1.50	38.78	3.33	52.42	99.70	0.47	0.0001
Size Intercept Sex (male)	54	65	172	0.44 (−0.31, 1.08) 0.09 (−0.10, 0.26)	1.41	7.43	17.11	3.97	69.29	99.21	0.30	0.0007
Survival Intercept Sex (male)	36	52	145	1.17 (−0.30, 2.64) −0.38 (−1.00, 0.17)	1.16	2.06	1.25	28.98	66.19	99.64	0.34	0.001

Bold values of *d* highlight 95% credible intervals that do not overlap zero, indicating significant phenotypic plasticity in that trait overall. The 95% CIs of the deviation in *d* due to sex (male) overlapped zero, indicating no significant difference in plasticity between the sexes, in all trait classes except cold resistance. Males showed a significantly smaller plastic response in cold resistance traits than did females. *N*_species_, *N*_papers_, *N*_exp_: number of species, papers, and independent experiments within papers, respectively. *I* ^2^: percentage of heterogeneity among effect sizes attributable to the grouping level (variance component) indicated by the subscript in each column, summing to *I* ^2^_total_; *R* ^2^_conditional_: variance explained by fixed and random factors; *R* ^2^_marginal_: variance explained by fixed factors (Nakagawa & Schielzeth, 2013).

Cold and heat resistance both improved following exposure to a thermal manipulation, consistent with our expectations. For development time, gene expression and longevity, the directions of effects also matched our expectations: faster development and higher gene expression were associated with warmer temperatures, and increased longevity was associated with colder temperatures. Mean Hedges’ *d* did not differ from zero for the size or survival trait classes (Table 2), likely due to many responses of opposing direction within these classes. Additionally, our moderator analyses showed that, for the survival trait class, the direction of *d* depended on the direction of the temperature manipulation (whether individuals were exposed to extreme cold or extreme heat) (Figure S4). Heterogeneity in the direction of plastic responses to temperature is also suggested by analysis of |*d*|, which found that mean absolute magnitudes of plastic responses were at least 50% greater than directional estimates, and up to 5 times greater for gene expression (Figure S1).

Heterogeneity of effect sizes within each trait class was generally high (*I* ^2^ *_total_* = 95.79—99.98%; Table 2). In most classes, a substantial proportion of this heterogeneity was attributable to differences between studies (*I* ^2^ *_paper_*) and experiments within studies (*I* ^2^ *_experiment_*), while phylogenetic relatedness (*I* ^2^ *_phylogeny_*) and species identity (*I* ^2^ *_species_*) accounted for little further heterogeneity in any trait class. The experimental design differences explored in our subsequent moderator analyses (different thermal manipulations, particular traits within trait classes, and different temperature deviations; Table S1) significantly affected estimates of *d* in few trait classes, and we fully explore these effects in the supplementary material.

Meta‐analyses of variance found little effect of temperature or sex on trait variability. Analysis of *lnCVR_treatment_*—the log ratio of coefficients of variation between each pairwise combination of treatment levels—found a significant effect of temperature on trait variance for development time, which was more variable in colder treatments (Table S2, Figure S2a); this pattern was significantly stronger for females than for males. The gene expression trait class also showed a significant sex difference in *lnCVR_treatment_*, although neither estimate differed significantly from zero (Table S2, Figure S2a). Sex‐specific *lnCVR_treatment_* estimates did not differ significantly for any other trait class (Table S2). Finally, analysis of *lnCVR_sex_*—the log ratio of coefficients of variation between males and females at each treatment level –found no effect of sex on trait variance in any trait class (Table S3, Figure S2b). Heterogeneity and the effects of moderator variables in these analyses are explored in the supplementary material (Figures S3–S8), along with tests for potential publication biases in all analyses (Tables S4, S5).

## DISCUSSION

How populations respond to environmental change is crucial to their persistence, particularly given the accelerating pace of contemporary climate change (Collins et al., 2013). Our theoretical models show that sex differences in phenotypic plasticity, genetic variation and the strength and direction of selection, can strongly affect adaptation and population persistence under environmental change. We predict that female‐biased phenotypic plasticity should promote population persistence for species that fall into the female demographic dominance category and for traits that have a weak cross‐sex genetic correlation. Sex differences in phenotypic plasticity are, on the other hand, more likely to hinder population persistence in species where the sexes contribute equally to parental investment and for traits with strong cross‐sex genetic correlations. Our meta‐analysis of sex‐specific plasticity from 258 invertebrate studies, spanning a broad range of life‐history and thermal response traits, found few systematic differences between the sexes in their plastic responses to temperature manipulations. However, many individual studies included in our meta‐analysis demonstrated strong sex differences in plastic responses to temperature. Below, we discuss biological and experimental factors that might drive variation in sexually dimorphic plasticity, their implications for extinction susceptibilities, as well as some future directions.

With the exception of cold resistance, where females were more plastic than males, the sexes did not systematically differ in their levels of phenotypic plasticity in response to different thermal treatments. Moreover, variation in plasticity did not systematically differ between the sexes. Our results are consistent with a recent meta‐analysis that found weak evidence for sex‐biased plasticity in thermal acclimation of ectothermic species (Pottier et al., 2021). The general result of our meta‐analysis suggests that consistently female‐biased phenotypic plasticity is unlikely to broadly mediate the challenges of changing thermal environments under climate change. Nevertheless, sex‐specific plasticity might be important in facilitating adaptation for specific traits and/or species. For example, while studies explicitly comparing trait genetic variances between sexes are relatively rare, greater genetic variances in males than females have been documented for some traits (see Wyman & Rowe, 2014), providing a context where female‐biased plasticity may be beneficial. Whether environmental change differentially affects the phenotypic optima for females and males, while largely unknown, should also affect whether or not sex‐biased plasticity is favourable. Presently, it is difficult to make generalisations about the scope of sexual dimorphism for trait genetic variances or rates of change in trait optima, and these gaps are clearly in need of empirical attention.

Sex differences in parental investment will also strongly affect the importance of sex‐biased plasticity, with female‐biased plasticity more likely to promote persistence when parental investment is also female‐biased (i.e. female demographic dominance). Although female‐biased parental investment is typical among animals (Kokko & Jennions, 2008), species vary in how much each sex invests in their offspring and both uniparental and biparental care have been reported in insect species (e.g. Suzuki, 2013). Recent studies have suggested that the mating system can affect sex‐specific thermal phenotypic plasticity (e.g. Bauer et al., 2021). Although monogamy is extremely rare in insects, many social insect species, including termites and cockroaches, are monogamous (e.g. Boomsma, 2009). While the species included in this study no doubt vary in parental investment, we are not aware of an example of male parental investment or mate provisioning, biparental care, or strict monogamy in the species included in this study. We expect that nearly all taxa will fall somewhere along the spectrum of female demographic dominance, though perhaps not matching the idealised form in which growth overwhelmingly depends on females (for further discussion, see Rankin & Kokko, 2007).

Previous theory has emphasised that positive cross‐sex genetic correlations in traits mediating fitness (r*_mf_* >0, as broadly observed for quantitative traits; Poissant et al., 2010, Matthews et al., 2019) should promote adaptation of both sexes, along with population persistence, provided directional selection in altered environments predominantly aligns between the sexes (see Berger et al., 2014; Bonduriansky & Chenoweth, 2009; Cally et al., 2019; Connallon, 2015; Connallon & Hall, 2016; Lande, 1980). The theory presented here suggests that female‐biased plasticity is more likely to promote population growth when r*_mf_* values are modest‐to‐low than when they are large and positive. As r*_mf_* approaches unity, sexual dimorphism in plasticity is expected to hinder population growth, particularly when the amount of genetic variation and the rates of change of trait optima are similar between the sexes. This effect arises because sex differences in plasticity, in these circumstances, generate conflicting patterns of directional selection between the sexes, despite parallel patterns of change in each sex's optimum. Stronger directional selection in the sex experiencing lower plasticity can displace the sex with higher plasticity from its optimum—an effect that is more apparent when the sexes are tightly genetically coupled via a strong and positive cross‐sex genetic correlation. Previous research has shown that cross‐sex genetic correlations, though generally positive, tend to be larger for morphological than physiological traits (Poissant et al., 2010). For example, r*_mf_* for body size is often near unity, and the lack of systematic sex differences in phenotypic plasticity for this trait class may, therefore, be beneficial in terms of population persistence. Among physiological trait categories, which tend to have weaker cross‐sex genetic correlations, female‐biased plasticity is perhaps likely to promote persistence. Previous estimates of r*_mf_* for cold tolerance suggest that cross‐sex genetic correlations may vary between r*_mf_* ~0.5 (Morgan & Mackay, 2006) and r*_mf_* ~1.0 (Hangartner et al., 2020) which provides context where systematically female‐biased plasticity (as we observed) may confer population benefits. Weaker cross‐sex genetic correlations in physiological traits might, therefore, also contribute to why cold and heat resistance showed more variability in sex differences than some of the other trait classes.

Our models clarify how sexually dimorphic phenotypic plasticity, the contributions of each sex to population demography, and interactions between selection and sex‐specific genetic variation can jointly affect population persistence in changing environments. A related question, that our models do not address, is when sex differences in plasticity are expected to evolve in the first place. From existing theory, we know that the evolution of phenotypic plasticity depends on: (i) the presence of genetic variation for plasticity, (ii) the relation between trait expression and fitness, (iii) the nature of spatiotemporal changes in the environment, (iv) costs of plasticity and (v) the predictability of environmental change (Crispo et al., 2010; Lande, 2014; Murren et al., 2015). Each of these factors may affect opportunities for sex‐specific plasticity to evolve. For example, the rate at which sexually dimorphic plasticity can evolve should depend on the amount of genetic variation for plasticity within each sex and the strength of genetic correlations for plasticity between the sexes. While genetic variation for plasticity appears to be substantial (Crispo et al., 2010; Murren et al. 2014; but see Charmantier et al., 2008), it is unclear how much of the variability is sex‐specific (e.g. Karan et al., 2007), and whether there is much evolutionary potential for evolving sex differences in plasticity. Fitness consequences of plasticity, as well as the predictability of the abiotic, biotic and social environments (e.g. Bradshaw, 1965; Snell Rood et al., 2010; Crispo et al., 2010; Lande, 2014, Scheiner, 2019; Kelly, 2019), may differ between the sexes, resulting in sex differences in directional selection that favour the evolution of sexually dimorphic plasticity. Additionally, the relative importance of these factors is likely to vary among traits, species and environments. Formal models for the evolutionary origin of sexually dimorphic plasticity would help clarify when and where we should expect to observe sex differences in plasticity, in general.

Importantly, experimental factors may also contribute to the lack of a general pattern of sex‐specific plasticity in our meta‐analysis. Indeed, differences between studies and experiments accounted for substantial variation around our meta‐analytic means. In particular, we found that how traits were measured, and which thermal treatment was applied, had a strong effect in some trait classes. For example, compared to acclimation, hardening, and rearing treatments, acute thermal treatments had opposite effects on both cold resistance and development time, possibly due to acute treatments being more stressful and providing little opportunity for thermal acclimation. However, experimental design factors did not explain substantial between‐study heterogeneity for most of the trait classes examined, suggesting that other differences in experimental approaches, statistical power, and study populations may contribute to the observed variation. Phylogeny accounted for little variation in our analyses, indicating that the evolution of sexual dimorphism in phenotypic plasticity is not driven by a shared evolutionary history. However, not all taxa were equally represented, so our analyses may have had limited statistical power to detect a phylogenetic signal.

Our meta‐analyses have focused on plasticity in relation to natural selection and extrinsic environmental (thermal) variation, yet it is important to acknowledge a large theoretical and empirical literature on sexually dimorphic plasticity relating to sexual selection (Bonduriansky, 2007; Garcia‐Roa et al., 2020; Stillwell et al., 2010). It has long been known that females and males experience different forms of selection on traits mediating mate competition and fertilisation success (Clutton‐Brock, 2007), which are often subject to stronger selection in males than females (Rohner & Blanckenhorn, 2018; Singh & Punzalan, 2018). Sexual selection is expected to favour the evolution of condition‐dependent (i.e. plastic) expression of sexually selected traits (Rowe & Houle, 1996), and because sexually selected traits are often more strongly expressed by males, their condition‐dependence will naturally lead to male‐biased plasticity for male‐elaborated secondary sexual traits (e.g. Bonduriansky, 2007; Bonduriansky & Rowe, 2007). Whether such observations can be generalised requires empirical tests assessing sex‐specific plasticity in traits that differ in the type and strength of selection acting on them.

Our meta‐analysis included a large number of studies (*n* = 258) spanning 205 species. Nevertheless, a closer look reveals three general limitations of the available data, and thus opportunities for future empirical work. First, while insects are strongly represented in our dataset (93% of studies), other phylogenetic groups are not. As we found few vertebrate (6%) and plant (8%) studies in our initial search, we only included invertebrate species in our database. Future work should prioritise the collection of thermal tolerance trait data, in particular, from non‐insect invertebrate, vertebrate and plant taxa. A second limitation is that almost all studies focus on thermal plasticity traits expressed during adult stages, particularly so for cold and heat resistance traits. Plasticity has repeatedly been shown to be life‐stage specific (e.g. Moghadam et al., 2019), which highlights the need for further attention to pre‐adult stages. Finally, perhaps the most important limitation of phenotypic plasticity studies is our limited knowledge of the fitness effects of plasticity, and whether it is generally adaptive. Straightforward inferences of adaptive plasticity are possible for some combinations of traits and environmental treatments. For example, plastic responses involving thermal resistance are likely to be adaptive in most cases, at least for acclimation treatments, which all consistently improve thermal resistance (Cossins & Bowler, 1987), although such responses may be associated with reduced performance under other environmental conditions (Kristensen et al., 2008; Sgrò et al., 2016). There may often be limits to this generalisation, however, as plastic responses to *extremely* stressful treatments often reduce performance, representing cases of maladaptive plasticity (Ghalambor et al., 2007). Yet, few studies actually estimate the effect of plasticity on fitness and thereby directly test whether plasticity is adaptive (e.g. Wilson & Franklin, 2002). Direct estimates of fitness effects of plasticity are needed to evaluate the adaptive implications of plasticity, and its consequences for population responses to climate changes. This goal should include assessments of sex‐specific fitness consequences of phenotypic plasticity.

## CONFLICTS OF INTEREST

All authors have agreed on submission of the current version of the article and there are no conflicts of interest that bias our work.

## AUTHOR CONTRIBUTIONS

SH, CS, IB and TC planned the study. SH collected the data, IB analyzed the data and TC developed the theory. SH, IB and TC wrote parts of the manuscript and CS revised earlier drafts. All authors read and approved the final draft of the manuscript.

### PEER REVIEW

The peer review history for this article is available at https://publons.com/publon/10.1111/ele.14005.

## Supporting information

Supplementary Material

Supplementary Material

## Data Availability

Data have been submitted to Dryad and is currently under review. DOI: https://doi.org/10.5061/dryad.7d7wm37x8
