# Supplementary material for: Sexual dimorphism in phenotypic plasticity and persistence under environmental change: An extension of theory and meta‐analysis of current data
Source: Ecol Lett. 2022 Mar 25;25(6):1550–65. doi: 10.1111/ele.14005 (PMC9311083; doi:10.1111/ele.14005)
Supplement: Supplementary file 1 — Supplementary Material [file ELE-25-1550-s002.docx]

**Supplementary tables**

**Table S1.** Levels of the moderator variables applied in each data subset. ‘Treatments applied’ describes the main classes of thermal manipulation used. For the subsets Cold resistance and Heat resistance, ‘Traits measured’ captures the difference between studies in their use of particular measures of resistance; for the remaining subsets, ‘Temperature direction’ differentiates between studies imposing cold stress, heat stress, or simply different (not necessarily ‘stressful’) thermal environments.

| Data subset | Traits measured | Treatments applied | Temperature direction |
| --- | --- | --- | --- |
| Cold resistance | CTmin  Recovery time  Supercooling point | Acclimation  Hardening  Rearing temperature  Acute (cold) |  |
| Development time^1^ |  | Rearing temperature  Acute (cold) | Extreme cold  No obvious extreme |
| Heat resistance | CTmax  Knockdown time  Knockdown temp | Acclimation  Hardening  Rearing temperature  Acute (heat) |  |
| Gene expression |  | Hardening  Rearing temperature  Acute (heat/cold) | Extreme cold  Extreme heat  No obvious extreme |
| Longevity |  | Acclimation  Hardening  Rearing temperature  Acute (heat/cold) | Extreme cold  Extreme heat  No obvious extreme |
| Size |  | Acclimation  Rearing temperature  Acute (cold) | Extreme cold  Extreme heat  No obvious extreme |
| Survival |  | Acclimation  Hardening  Rearing temperature  Acute (heat/cold) | Extreme cold  Extreme heat  No obvious extreme |

^1^ The variable ‘Temperature direction was redundant with respect to ‘Treatments applied’ for the Development time data subset, so only ‘Treatments applied' was modelled.

**Table S2.** Meta-analytic mean estimates and 95% credible intervals of *lnCVR_treatment_* (the log ratio of coefficients of variation between each pairwise combination of treatment levels), for females (Intercept) and males across seven trait classes. Positive values of *lnCVR* indicate greater variation in treatment 2 than in treatment 1; for the classes cold resistance, gene expression, and heat resistance treatment 2 was the colder treatment, while for the remaining classes treatment 2 was the warmer treatment (see Methods). Effects in bold highlight 95% credible intervals that do not overlap zero. N_species_, N_papers_, N_exp_: number of species, papers, and independent experiments within papers, respectively. I^2^: percentage of heterogeneity among effect sizes attributable to the grouping level (variance component) indicated by the subscript in each column, summing to I^2^_total_; R^2^_conditional_: variance explained by fixed and random factors; R^2^_marginal_: variance explained by fixed factors (Nakagawa & Schielzeth 2013).

|  | *N_species_* | *N_papers_* | *N_exp_* | *lnCVR* (95% CI) | I^2^_species_ (%) | I^2^_phylogeny_ (%) | I^2^_paper_ (%) | I^2^_experiment_ (%) | I^2^_residual_ (%) | I^2^_total_ (%) | R^2^_conditional_ | R^2^_marginal_ |
| --- | --- | --- | --- | --- | --- | --- | --- | --- | --- | --- | --- | --- |
| Cold resistance  Intercept  Sex (male) | 39 | 34 | 103 | 0.07 (-0.10, 0.26)  -0.06 (-0.17, 0.04) | 1.89 | 3.01 | 5.50 | 1.40 | 74.98 | 86.78 | 0.14 | 0.003 |
| Development time  Intercept  Sex (male) | 67 | 69 | 158 | **-0.14 (-0.27, -0.03)**  **0.07 (0.02, 0.11)** | 5.18 | 2.15 | 4.59 | 3.28 | 81.37 | 96.57 | 0.16 | 0.003 |
| Heat resistance  Intercept  Sex (male) | 25 | 20 | 103 | -0.19 (-0.62, 0.18)  0.01 (-0.08, 0.11) | 5.88 | 29.38 | 4.52 | 0.99 | 45.95 | 86.72 | 0.47 | 0.001 |
| Gene expression  Intercept  Sex (male) | 12 | 18 | 84 | 0.02 (-0.38, 0.44)  **-0.13 (-0.25, -0.01)** | 3.76 | 3.30 | 4.32 | 11.44 | 66.76 | 89.58 | 0.26 | 0.003 |
| Longevity  Intercept  Sex (male) | 92 | 99 | 179 | -0.08 (-0.18, 0.04)  -0.01 (-0.06, 0.03) | 4.21 | 1.83 | 5.29 | 1.19 | 83.86 | 96.37 | 0.13 | 0.0004 |
| Size  Intercept  Sex (male) | 54 | 65 | 172 | -0.02 (-0.12, 0.07)  0.01 (-0.03, 0.06) | 2.94 | 2.49 | 7.74 | 1.03 | 79.55 | 93.75 | 0.30 | 0.001 |
| Survival  Intercept  Sex (male) | 36 | 52 | 145 | -0.04 (-0.42, 0.30)  -0.08 (-0.22, 0.03) | 2.18 | 2.00 | 12.18 | 18.91 | 60.97 | 96.21 | 0.37 | 0.001 |

**Table S3.** Meta-analytic mean estimates and 95% credible intervals of *lnCVR_sex_* (the log ratio of coefficients of variation between males and females at the same treatment level) across seven trait categories. Positive values of *lnCVR* indicate greater variation in females than in males. Effects in bold highlight 95% credible intervals that do not overlap zero. N_species_, N_papers_, N_exp_: number of species, papers, and independent experiments within papers, respectively. I^2^: percentage of heterogeneity among effect sizes attributable to the grouping level (variance component) indicated by the subscript in each column, summing to I^2^_total_; R^2^: estimate of model goodness-of-fit.

|  | *N_species_* | *N_papers_* | *N_exp_* | *lnCVR* (95% CI) | I^2^_species_ (%) | I^2^_phylogeny_ (%) | I^2^_paper_ (%) | I^2^_experiment_ (%) | I^2^_residual_ (%) | I^2^_total_ (%) | R^2^ |
| --- | --- | --- | --- | --- | --- | --- | --- | --- | --- | --- | --- |
| Cold resistance | 39 | 34 | 103 | 0.04 (-0.11, 0.19) | 2.55 | 5.14 | 3.02 | 2.33 | 63.96 | 77.00 | 0.19 |
| Development time | 67 | 71 | 165 | -0.05 (-0.12, 0.02) | 1.87 | 2.24 | 2.05 | 1.53 | 82.13 | 89.82 | 0.09 |
| Heat resistance | 25 | 21 | 107 | -0.03 (-0.25, 0.18) | 15.45 | 16.71 | 4.40 | 1.64 | 30.82 | 69.02 | 0.54 |
| Gene expression | 12 | 19 | 88 | 0.02 (-0.17, 0.23) | 1.74 | 2.39 | 2.61 | 1.38 | 64.04 | 72.15 | 0.11 |
| Longevity | 92 | 99 | 180 | -0.06 (-0.14, 0.03) | 2.65 | 2.56 | 4.98 | 1.91 | 80.27 | 92.37 | 0.13 |
| Size | 54 | 66 | 176 | 0.06 (-0.07, 0.17) | 4.84 | 5.80 | 19.04 | 1.44 | 60.56 | 91.67 | 0.34 |
| Survival | 36 | 53 | 158 | -0.07 (-0.26, 0.16) | 2.18 | 2.50 | 18.65 | 5.30 | 61.25 | 89.88 | 0.32 |

**Table S4.** Egger’s regression models for asymmetry in ‘funnel plots’ of an estimate’s residual error and its precision (inverse of standard error). Effects in bold highlight estimates differing significantly from zero at alpha = 0.05 (indicating asymmetry).

|  | **Hedges' *d*** | | | ***lnCVR_treatment_*** | | | ***lnCVR_sex_*** | | |
| --- | --- | --- | --- | --- | --- | --- | --- | --- | --- |
|  | *ß ± SE* | *Z* | *p* | *ß ± SE* | *Z* | *p* | *ß ± SE* | *Z* | *p* |
| Cold resistance | -0.002 ± 0.143 | -0.01 | 0.99 | 0.030 ± 0.027 | 1.11 | 0.27 | -0.005 ± 0.031 | -0.17 | 0.87 |
| Development time | 0.009 ± 0.154 | 0.06 | 0.95 | -0.006 ± 0.012 | -0.47 | 0.64 | -0.0002 ± 0.017 | -0.01 | 0.99 |
| Heat resistance | 0.024 ± 0.060 | 0.40 | 0.69 | 0.023 ± 0.024 | 0.95 | 0.34 | -0.003 ± 0.023 | -0.14 | 0.89 |
| Gene expression | 0.002 ± 0.197 | 0.01 | 0.99 | **-0.063 ± 0.030** | **-2.06** | **0.04** | -0.019 ± 0.042 | -0.45 | 0.65 |
| Longevity | 0.005 ± 0.051 | 0.10 | 0.92 | 0.005 ± 0.012 | 0.39 | 0.69 | -0.001 ± 0.018 | -0.04 | 0.96 |
| Size | 0.004 ± 0.042 | 0.10 | 0.92 | 0.007 ± 0.012 | 0.59 | 0.56 | 0.001 ± 0.016 | 0.06 | 0.95 |
| Survival | 0.007 ± 0.145 | 0.05 | 0.96 | **-0.083 ± 0.033** | **-2.52** | **0.01** | 0.004 ± 0.034 | 0.11 | 0.91 |

**Table S5.** Meta-analytic mean estimates and 95% credible intervals of Hedges’ *d*, *lnCVR_treatment_* and *lnCVR_sex_*, across seven trait categories, including the moderator variables year of publication (centered and mean standardised) and the interaction between year and sex to test for changes in effect size over time. Effects in bold highlight 95% credible intervals that do not overlap zero.

|  | *Hedges’ d* | *lnCVR_treatment_* | *lnCVR_sex_* |
| --- | --- | --- | --- |
| Cold resistance  Intercept  Sex (male)  Year  Sex*Year | **1.88 (1.10, 2.81)**  **-0.83 (-1.50, -0.25)**  **1.01 (0.24, 1.74)**  -0.39 (-1.08, 0.19) | 0.09 (-0.09, 0.26)  -0.07 (-0.16, 0.05)  0.02 (-0.12, 0.17)  0.09 (-0.02, 0.20) | 0.04 (-0.11, 0.20)  -0.03 (-0.13, 0.07) |
| Development time  Intercept  Sex (male)  Year  Sex*Year | **9.16 (6.20, 11.61)**  -0.48 (-1.11, 0.13)  -2.09 (-4.63, 0.10)  0.06 (-0.61, 0.66) | **-0.14 (-0.28, -0.02)**  **0.07 (0.02, 0.11)**  -0.08 (-0.17, 0.01)  0.02 (-0.02, 0.07) | -0.05 (-0.12, 0.02)  0.01 (-0.04, 0.05) |
| Heat resistance  Intercept  Sex (male)  Year  Sex*Year | **0.61 (0.07, 1.11)**  0.07 (-0.16, 0.32)  0.10 (-0.20, 0.42)  0.07 (-0.19, 0.29) | -0.19 (-0.59, 0.19)  0.01 (-0.08, 0.10)  0.04 (-0.08, 0.15)  0.01 (-0.08, 0.10) | -0.03 (-0.21, 0.16)  0.07 (-0.02, 0.15) |
| Gene expression  Intercept  Sex (male)  Year  Sex*Year | **2.21 (0.25, 4.28)**  -0.07 (-0.87, 0.72)  0.26 (-1.28, 1.67)  -0.09 (-0.83, 0.73) | 0.01 (-0.41, 0.44)  **-0.12 (-0.24, -0.003)**  -0.11 (-0.37, 0.18)  **0.17 (0.06, 0.29)** | 0.02 (-0.17, 0.20)  -0.08 (-0.24, 0.08) |
| Longevity  Intercept  Sex (male)  Year  Sex*Year | **2.14 (1.30, 2.90)**  -0.002 (-0.22, 0.19)  0.14 (-0.29, 0.56)  -0.06 (-0.27, 0.13) | -0.08 (-0.18, 0.05)  -0.01 (-0.06, 0.03)  -0.03 (-0.09, 0.02)  0.03 (-0.02, 0.08) | -0.06 (-0.15, 0.03)  -0.04 (-0.09, 0.01) |
| Size  Intercept  Sex (male)  Year  Sex*Year | 0.15 (-0.71, 0.89)  0.09 (-0.09, 0.25)  **-0.48 (-0.73, -0.22)**  -0.10 (-0.29, 0.07) | -0.001 (-0.11, 0.11)  0.01 (-0.03, 0.05)  **0.08 (0.03, 0.14)**  **-0.05 (-0.10, -0.01)** | 0.06 (-0.05, 0.19)  -0.02 (-0.09, 0.04) |
| Survival  Intercept  Sex (male)  Year  Sex*Year | 1.15 (-0.23, 2.45)  -0.37 (-0.98, 0.19)  **0.90 (0.01, 1.82)**  **-0.73 (-1.33, -0.14)** | -0.04 (-0.40, 0.32)  -0.08 (-0.20, 0.06)  -0.08 (-0.29, 0.15)  0.07 (-0.05, 0.19) | -0.07 (-0.29, 0.15)  0.01 (-0.13, 0.15) |

**Supplementary figures**

**
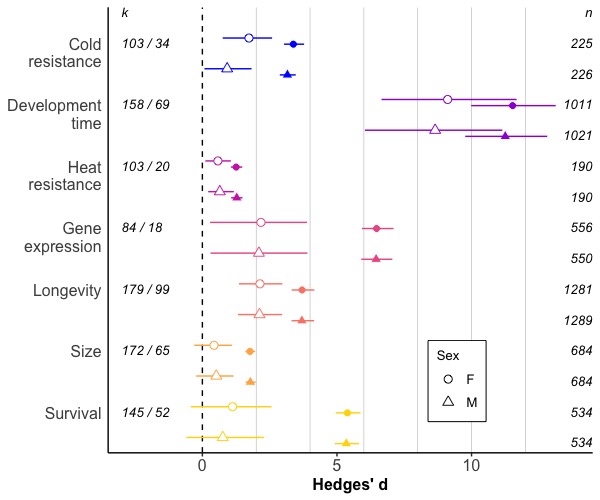
**

**Figure S1.** Meta-analytic mean estimates and 95% credible intervals of the absolute value of Hedges’ *d* (open symbols) and |*d*| (filled symbols) across seven trait categories for females (circles) and males (triangles); *k* indicates the number of independent experiments / papers in each category from which *n* effect sizes were extracted for each sex. Note that this figure presents the same mean estimates as in Figure 3A, but excludes the individual estimates to allow for greater visual clarity of the mean effects.

**
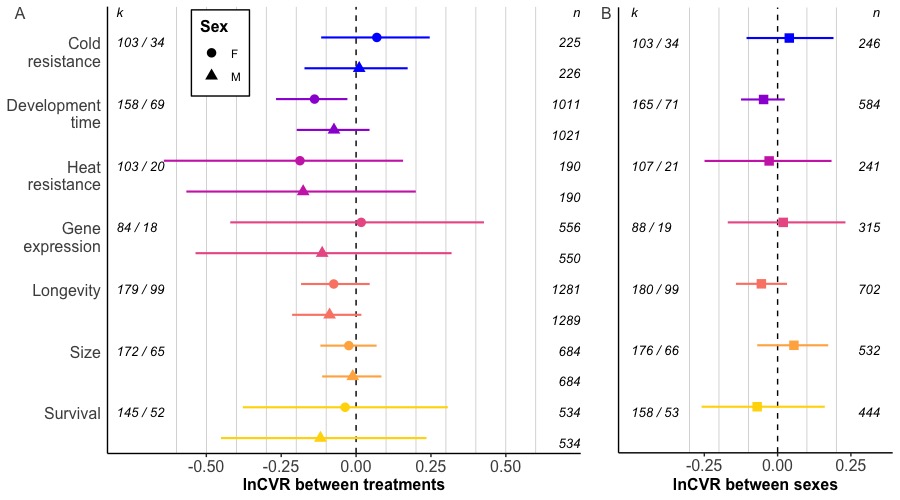
**

**Figure S2.** Meta-analytic mean estimates and 95% credible intervals of **A**) *lnCVR_treatment_* (the log ratio of coefficients of variation between each pairwise combination of treatment levels) for females (circles) and males (triangles), and **B**) *lnCVR_sex_*, (the log ratio of coefficients of variation between females and males at the same treatment level) across seven trait categories); *k* indicates the number of independent experiments / papers in each category from which *n* effect sizes were extracted for each sex. In (A), positive values indicate greater variation in treatment 2 than treatment 1; for the categories cold resistance, gene expression and heat resistance treatment 2 was the colder treatment, while for the remaining categories treatment 2 was the warmer treatment (see Methods). In (B), positive values indicate greater variation among females than males.

**
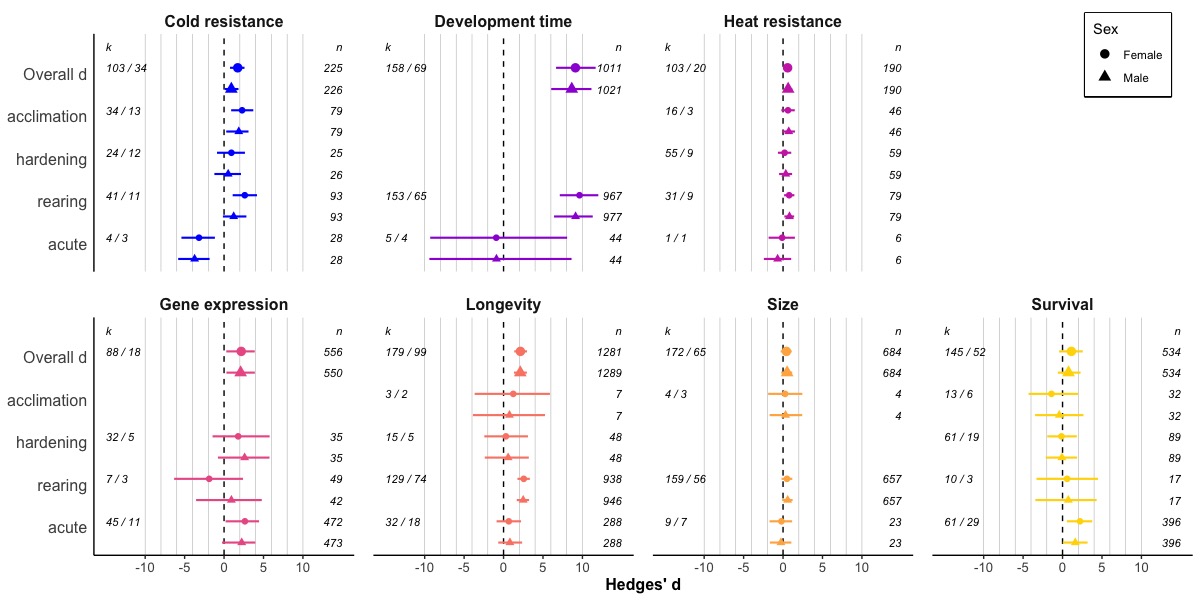
**

**Figure S3.** Meta-analytic mean estimates and 95% credible intervals of Hedges’ *d* for the moderator variable ‘Treatment Applied’.

**
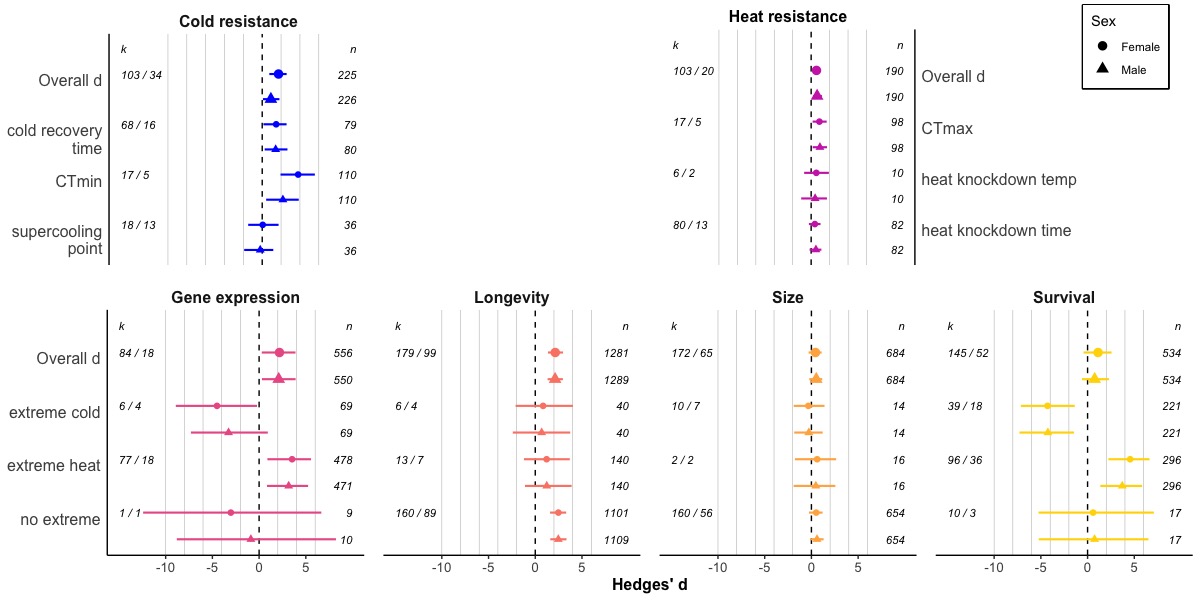
**

**Figure S4.** Meta-analytic mean estimates and 95% credible intervals of Hedges’ *d* for the moderator variables ‘Traits measured’ (top row) and ‘Temperature direction’ (bottom row).

**
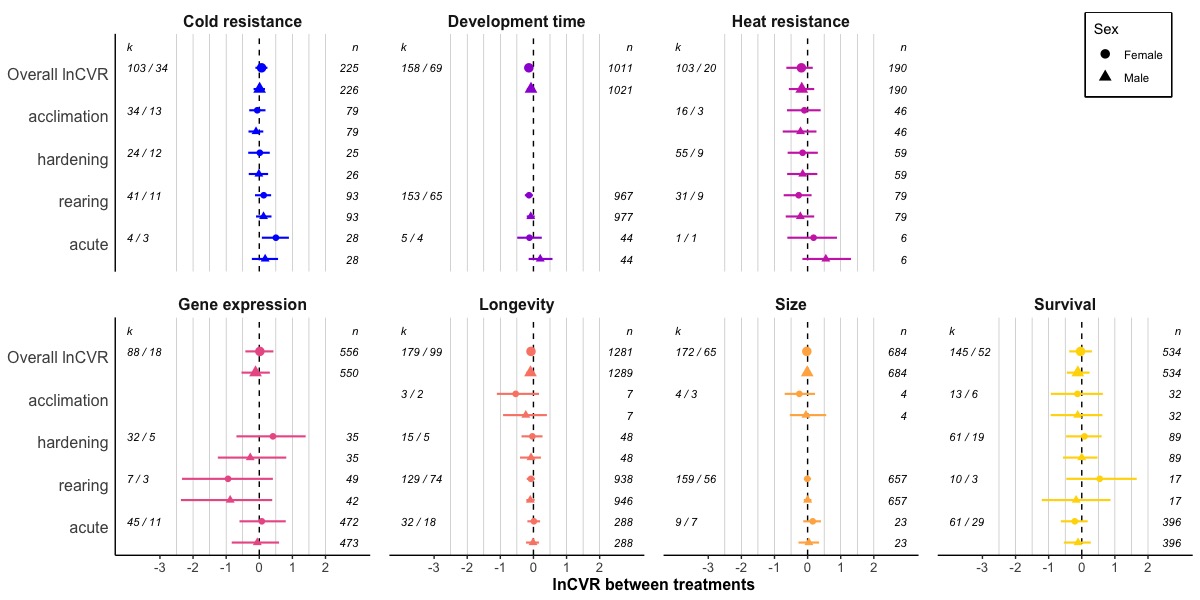
**

**Figure S5.** Meta-analytic mean estimates and 95% credible intervals of *lnCVR_treatment_* (the log ratio of coefficients of variation between each pairwise combination of treatment levels) for the moderator variable ‘Treatment Applied’. Positive estimates indicate greater trait variability in treatment 2 than treatment 1 (see text for treatment levels for each trait class).


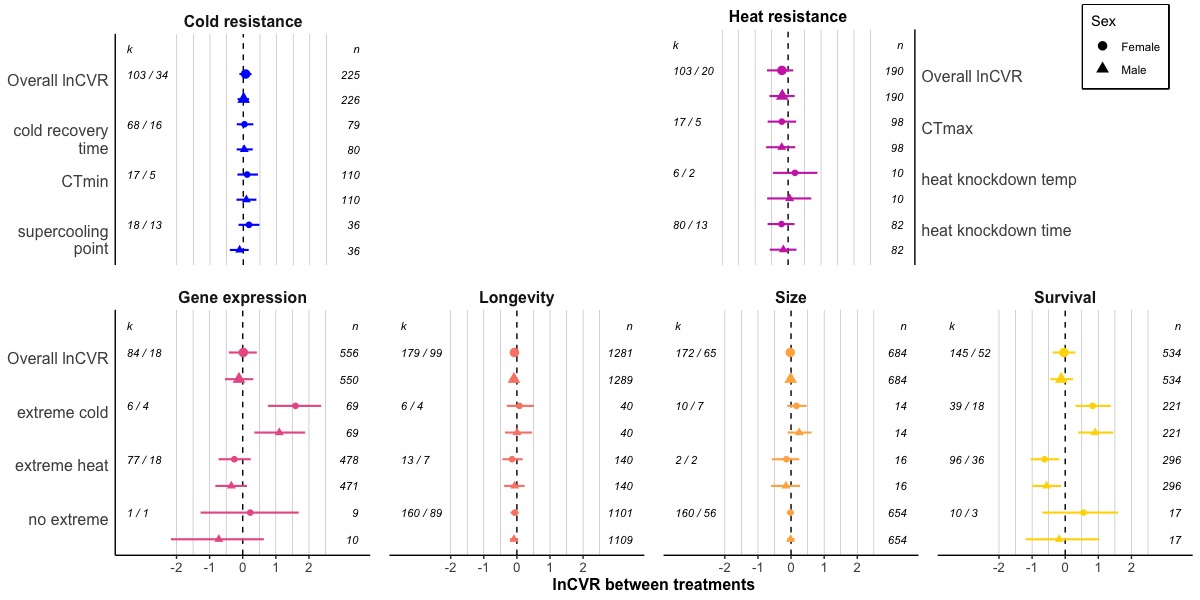


**Figure S6.** Meta-analytic mean estimates and 95% credible intervals of *lnCVR_treatment_* (the log ratio of coefficients of variation between each pairwise combination of treatment levels) for the moderator variables ‘Traits measured’ (top row) and ‘Temperature direction’ (bottom row). Positive estimates indicate greater trait variability in treatment 2 than treatment 1 (see text for treatment levels for each trait class).

**
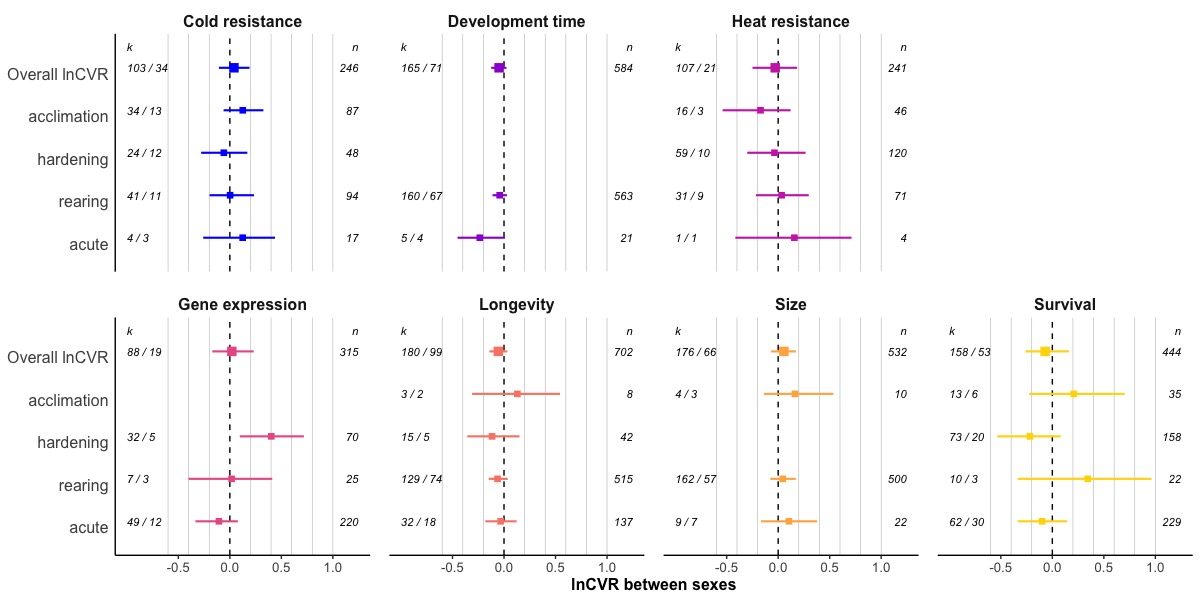
**

**Figure S7.** Meta-analytic mean estimates and 95% credible intervals of *lnCVR_sex_* (the log ratio of coefficients of variation between females and males) for the moderator variable ‘Treatment Applied’. Positive estimates indicate greater trait variability in females than males at a given treatment level.

**
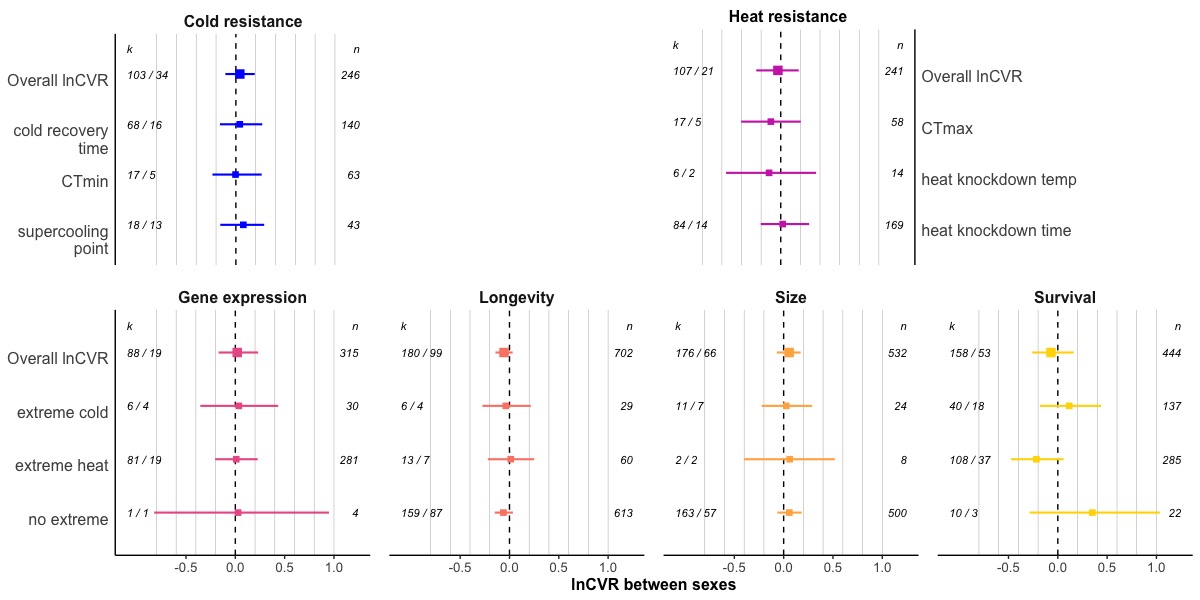
**

**Figure S8.** Meta-analytic mean estimates and 95% credible intervals of *lnCVR_sex_* (the log ratio of coefficients of variation between females and males) for the moderator variables ‘Traits measured’ (top row) and ‘Temperature direction’ (bottom row). Positive estimates indicate greater trait variability in females than males at a given treatment level.

**
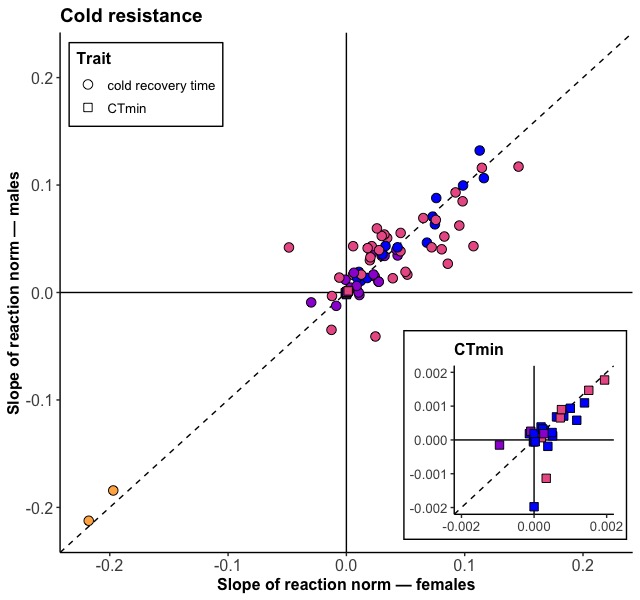

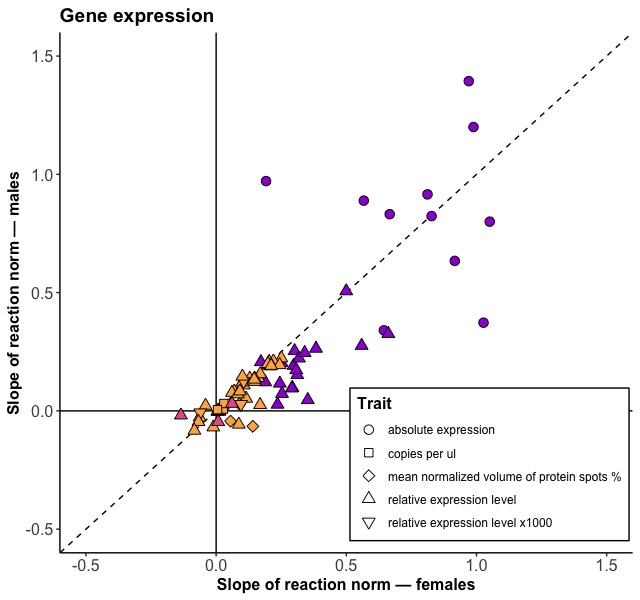

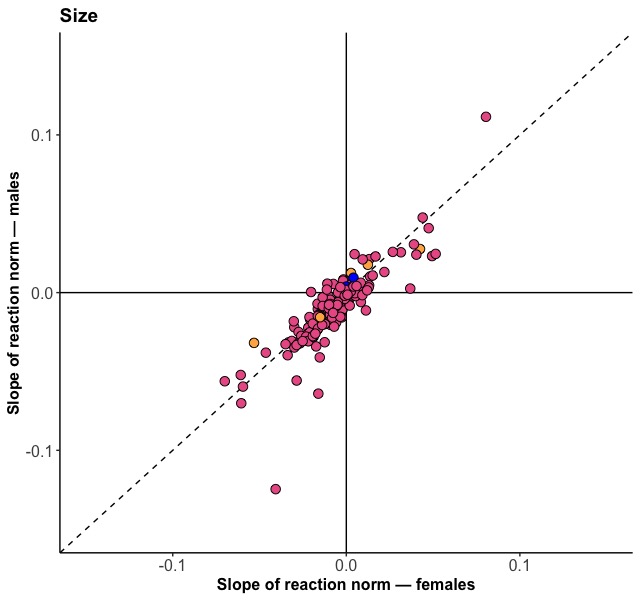
**

**
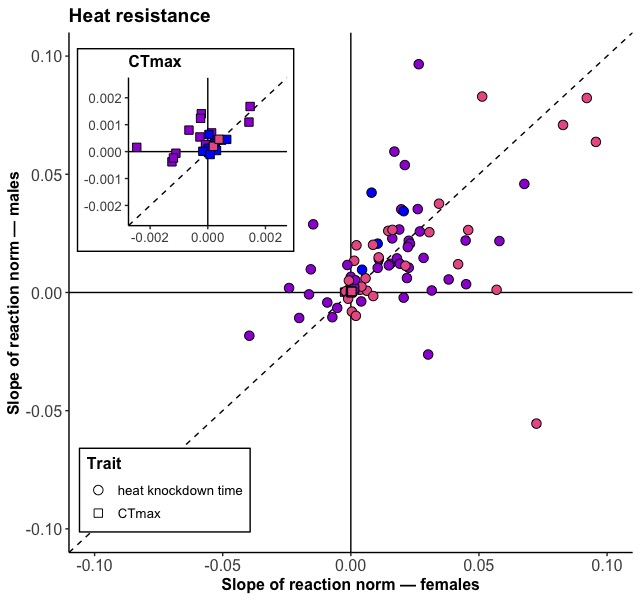

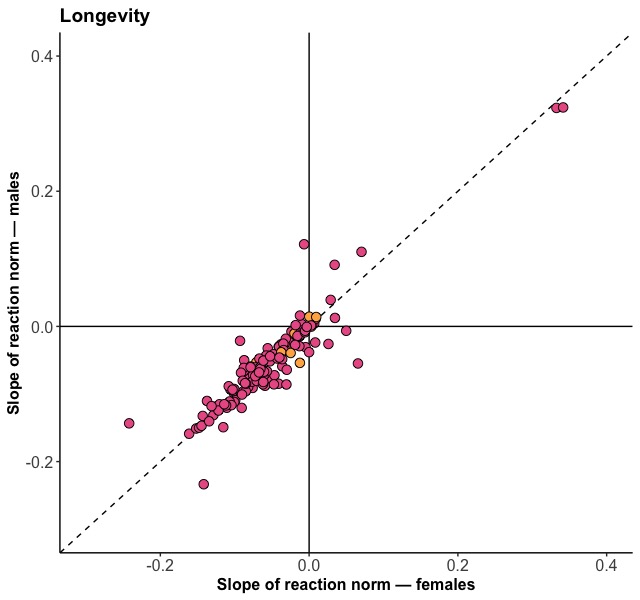

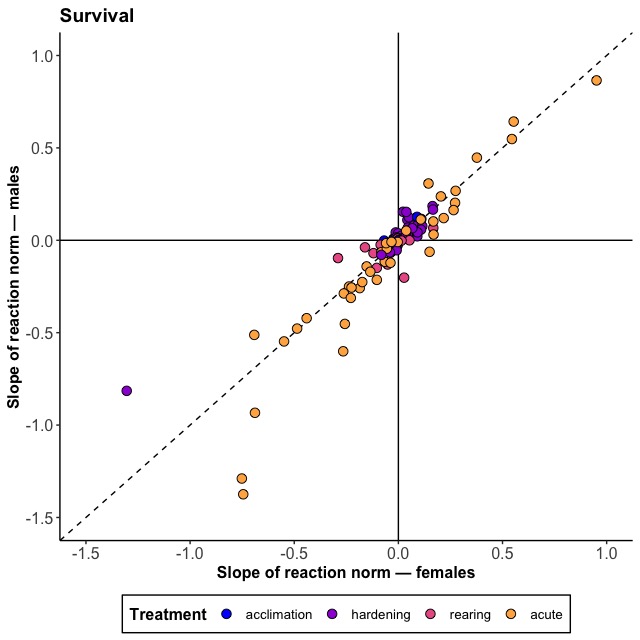
**

**Figure S9.** Scatter plots of the slopes of female and male reaction norms (mean standardized measures of cold resistance, heat resistance, gene expression, longevity, size and survival regressed against temperature) measured for a subset of experiments included in the meta-analyses. Colours indicate the treatment class, and symbols indicate different trait measures used in studies of cold resistance, heat resistance, and gene expression. Inset plots show cold resistance measured as CTmin, and heat resistance measured as CTmax, as the scale of these measures was much smaller than the scale for cold recovery time or heat knockdown time.
