# Supplementary material for: Sexual dimorphism in phenotypic plasticity and persistence under environmental change: An extension of theory and meta‐analysis of current data
Source: Ecol Lett. 2022 Mar 25;25(6):1550–65. doi: 10.1111/ele.14005 (PMC9311083; doi:10.1111/ele.14005)
Supplement: Supplementary file 2 — Supplementary Material [file ELE-25-1550-s001.docx]

**SUPPLEMENTARY METHODS**

**Extended theoretical models of population persistence**

Following Chevin et al. (2010), we focused on a single trait that mediates adaptation and population dynamics in a changing environment. Expression of the trait in a female and male, respectively, follows the following functions:

|  | $z_{f}=a_{f}+b_{f}\varepsilon+e_{f}$ | (1a), |
| --- | --- | --- |

and

|  | $z_{m}=a_{m}+b_{m}\varepsilon+e_{m}$ | (1b), |
| --- | --- | --- |

where *a_j_* refers to the genetic breeding value for the *j*th sex (*j* = {*f*, *m*}, where *f* and *m* refer to females and males, respectively), *b_j_* is the reaction norm for the trait (phenotypic plasticity in our model), *ε* is the focal environmental variable (*ε* = 0 in the reference environment), and *e_j_* captures residual environmental variation that is not explained by *ε*. As in Chevin et al. (2010), we assume that plasticity is fixed within the population (*b_f_* and *b_m_* are constant), and that *a_j_* and *e_j_* are independent random variables within a given sex. Thus, the mean and variance of the trait within the *j*th sex are given by $\bar{z}_{j}=\bar{a}_{j}+b_{j}\varepsilon$ and $V_{P,j}=V_{A,j}+V_{E,j}$, respectively, where *V_A,j_* is the additive genetic variance of the trait, and *V_E,j_* is the residual variance; overbars denote averages.

Fitness for the *j*th sex follows a Gaussian function of trait expression and plasticity:

|  | $W_{j}\propto\exp\left( -\frac{\left( \theta_{j}-z_{j} \right)^{2}}{2\omega_{s,j}^{2}}-\frac{b_{j}^{2}}{2\omega_{b,j}^{2}} \right)$ | (2), |
| --- | --- | --- |

where *θ_j_* is the optimal expression of the trait, $\omega_{s,j}^{2}$ and $\omega_{b,j}^{2}$ are positive constants that define (respectively) the fitness costs of non-optimal trait expression, and the cost of phenotypic plasticity. Assuming that the trait is normally distributed within the population, mean fitness of the *j*th sex becomes:

|  | $\bar{W}_{j}\propto\sqrt{\gamma_{j}\omega_{s,j}^{2}}\exp\left( -\frac{\gamma_{j}}{2}\left( \theta_{j}-\bar{z}_{j} \right)^{2}-\frac{b_{j}^{2}}{2\omega_{b,j}^{2}} \right)$ | (3), |
| --- | --- | --- |

where $\gamma_{j}=\left( \omega_{s,j}^{2}+V_{P,j} \right)^{-1}$ represents the strength of stabilizing selection near the optimum of the *j*th sex.

To evaluate how patterns of sex-specific adaptation in our models influence extinction susceptibility, we considered two idealized models of intrinsic population growth, each a function of female and male adaptation to the environmental change. As in previous evolutionary models of population persistence (see Lynch and Lande 1993; Lande and Shannon 1996; Chevin et al. 2010; Bell 2017), we focused on the intrinsic growth rate (*r*), rather than density-dependent growth, because *r* determines whether or not a population at low density will grow or decline. It is, therefore, a good predictor for extinction vs. persistence. In our first idealized scenario, population growth is entirely determined by adaptation in females – a scenario referred to as “female demographic dominance” (Harts et al. 2014). For this case, we modelled the intrinsic population growth rate as:

|  | $r=r_{\max}-\frac{\gamma_{f}}{2}\left( \theta_{f}-\bar{z}_{f} \right)^{2}$ | (4), |
| --- | --- | --- |

where $r_{\max}=r_{0}-b_{f}^{2}/\left( 2\omega_{b,f}^{2} \right)$ represents the intrinsic growth rate of a well-adapted population in which the female trait mean matches the female optimum, and *r*_0_ is the intrinsic growth rate of a well-adapted population where there is no cost of plasticity (see Chevin et al. 2010).

In our second idealized scenario, population growth is symmetrically impacted by the adaptation of females and males, and in this case, we modelled the intrinsic population growth rate as:

|  | $r=r_{\max}-\frac{\gamma_{f}}{4}\left( \theta_{f}-\bar{z}_{f} \right)^{2}-\frac{\gamma_{m}}{4}\left( \theta_{m}-\bar{z}_{m} \right)^{2}$ | (5), |
| --- | --- | --- |

where $r_{\max}=r_{0}-b_{f}^{2}/\left( 4\omega_{b,f}^{2} \right)-b_{m}^{2}/\left( 4\omega_{b,m}^{2} \right)$ represents the intrinsic growth rate of a well-adapted population in which the trait means of both sexes match their optima, and *r*_0_ is the intrinsic growth rate of a well-adapted population where there are no costs of plasticity. Note that eqs. (4) and (5) become equivalent when there are no sex differences in adaptation or the cost of plasticity. For most species (*i.e.*, excluding rare taxa with sex role reversals), the sensitivity of population dynamics to female and male adaptation is likely to follow a continuous spectrum between the extremes that are outlined in eq. (4) and eq. (5). Nevertheless, these bookend cases provide useful limits within which nearly all other biological cases of interest will be bound (for further possibilities, see Rankin and Kokko 2007).

We model sex-specific adaptation and growth in response to two scenarios of environmental change as follows. Under a linear (directional) change in the environment, $\frac{\partial\varepsilon\left( t \right)}{\partial t}=\eta$ represents the rate of change of the environment and $\frac{\partial\theta_{j}\left( t \right)}{\partial t}=B_{j}\eta$ represents the rate of change of the optimum for the *j*th sex (see Chevin et al. 2010). Under cyclic change in the environment, the focal environmental state follows a sine function over time, $\varepsilon\left( t \right)=A\sin\left( ct \right)$, where *A* is the amplitude of the environmental change, and *c* is a positive constant determining the period of each cycle. The sex-specific optimum for the jth sex follows the function: $\theta_{j}\left( t \right)=B_{j}A\sin\left( ct \right)$. Following Chevin et al. (2010), we assume that environmental change alters the optimum expression of the trait for each sex (with no effect on other aspects of the fitness landscape) and quantitative genetic parameters (variances and genetic correlations) remain constant over time. The rates of change of the female and male trait means will be:

|  | $\frac{\partial\bar{z}_{f}\left( t \right)}{\partial t}=\frac{\partial\bar{a}_{f}\left( t \right)}{\partial t}+b_{f}\frac{\partial\varepsilon\left( t \right)}{\partial t}=\frac{1}{2}\left( V_{A,f}\frac{\partial\ln\left( \bar{W}_{f} \right)}{\partial\bar{a}_{f}}+r_{mf}\sqrt{V_{A,f}V_{A,m}}\frac{\partial\ln\left( \bar{W}_{m} \right)}{\partial\bar{a}_{m}} \right)+b_{f}\frac{\partial\varepsilon\left( t \right)}{\partial t}$ | (6a), |
| --- | --- | --- |

and

|  | $\frac{\partial\bar{z}_{m}\left( t \right)}{\partial t}=\frac{\partial\bar{a}_{m}\left( t \right)}{\partial t}+b_{m}\frac{\partial\varepsilon\left( t \right)}{\partial t}=\frac{1}{2}\left( V_{A,m}\frac{\partial\ln\left( \bar{W}_{m} \right)}{\partial\bar{a}_{m}}+r_{mf}\sqrt{V_{A,f}V_{A,m}}\frac{\partial\ln\left( \bar{W}_{f} \right)}{\partial\bar{a}_{f}} \right)+b_{m}\frac{\partial\varepsilon\left( t \right)}{\partial t}$ | (6b), |
| --- | --- | --- |

where *r_mf_* is the cross-sex additive genetic correlation of the trait (see Lande 1980; Connallon and Hall 2016).

For each model of environmental change, the evolutionary dynamics of female and male traits eventually converge to a “steady-state” in which: (1) the evolutionary lag between trait mean and optimum remains constant over time (values of $\theta_{f}-\bar{z}_{f}$ and $\theta_{m}-\bar{z}_{m}$ eventually become constant over time in the directional change model), or (2) the evolutionary lag follows a predictable cycle (values of $\theta_{f}-\bar{z}_{f}$ and $\theta_{m}-\bar{z}_{m}$ eventually follow a steady-state cycle). Once the steady-state is reached, we calculated the equilibrium (steady-state) intrinsic growth rate of the population ($r_{eq.}$) under the directional change model, and the average growth rate ($\bar{r}$) across a full steady-state cycle of the cyclic change model, *i.e.*:

|  | $\bar{r}=\frac{1}{\tau}\int_{0}^{\tau} r\left( t \right)dt$ | (7), |
| --- | --- | --- |

where *r*(*t*) is the growth rate at time t, and t is the length of a complete cycle ($\tau=2\pi/c$ in our model) (see Crow and Kimura 1970, p. 9; Lande and Shannon 1996). For either scenario, the population will persist in the long run when $r_{eq.}$ or $\bar{r}$ is positive; the population will go extinct when $r_{eq.}$ or $\bar{r}$ is negative.

**Meta-analyses of sex-specific phenotypic plasticity**

***Literature search and inclusion criteria.*** We searched Google Scholar for the keywords “thermal tolerance”, “thermal resistance”, “thermal treatment”, “temperature treatment”, “hardening treatment”, “acclimation treatment”, and “thermal stress”, in combination with either “sex” or “females and males”. We added any studies included in the analysis of Teder and Tammaru (2005) that used thermal treatments. We screened the results of these searches by excluding journals outside of evolution, ecology, entomology, or agriculture, and scanning titles and abstracts for obvious irrelevance (e.g., non-invertebrate study organism). We read the entirety of the remaining 306 studies, and excluded a further 48 studies that did not meet our inclusion criteria:

1. Invertebrate study organism
2. Experimental study manipulating temperature – at least two thermal treatments
3. Phenotypic performance (heat or cold tolerance, survival, longevity, development time, size, or gene expression) measured for each thermal treatment
4. Both males and females measured in each treatment
5. Sex-specific means, standard deviations, and sample sizes reported for phenotypic measures from all treatment levels

Thermal treatments were categorized as hardening (short-term, minutes to hours, pre-exposure to a thermal treatment, before trait measurement), acclimation (long-term pre-exposure, days to weeks), rearing (manipulation of thermal conditions during the whole of development) or stress (exposure to extreme, acute thermal conditions during trait measurement) treatments. We excluded 6 studies that used other treatments, such as comparison between different fluctuating temperature regimes or different rates of temperature change, as these were too few to comprise a separate treatment category and could not easily be assigned to any of the four main treatment categories.

Phenotypic traits were grouped into seven broad classes: measures of cold resistance, heat resistance, development time, gene expression, longevity, size, and survival, which were the most common trait classes found in the available studies. We excluded 21 studies that measured phenotypic traits outside of these classes, as too few effects were available per trait. Each trait class included several traits, as different studies measured these aspects of performance differently. For example, heat resistance was measured alternatively as time until knockdown, temperature at which knockdown occurred, or CT_max_.

***Data extraction and coding.*** From each experiment within a study, we extracted group means and their associated errors and sample sizes for each sex and thermal treatment level. Data were reported in text, tabular, or graphical format. We used ImageJ (Rasband 1997, Abramoff et al. 2004) to extract means and errors from graphics. When a range of sample sizes was reported (e.g., “10 – 12 replicates per group”) we used the lowest value.

***Effect size calculation.*** For the formal meta-analyses we calculated *d* following Koricheva et al. (2013, p. 62) using the formula:

$d=\frac{\bar{x}_{1}- \bar{x}_{2}}{\sqrt{\frac{\left( n_{1}-1 \right){s_{1}}^{2}+ \left( n_{2}-1 \right){s_{2}}^{2}}{n_{1}+ n_{2}-2}}} J$ ,

where $\bar{x}$*_1_* , $\bar{x}$*_2_* , *s_1_* , *s_2_* , and *n_1_* , *n_2_* , are the respective means, standard deviations and sample sizes of treatment groups 1 and 2, and *J* is a correction for small sample size:

$$J=1- \frac{3}{4\left( n_{1}+ n_{2}-2 \right)-1}$$

(Hedges 1981, Koricheva et al. 2013 p. 62). The sampling error variance of *d* was calculated following the modified method of Hedges (1982):

$V=\left( 1- \frac{3}{4\left( n_{1}+ n_{2}-2 \right)-1} \right)^{2}\times\left( \frac{n_{1}+ n_{2}-2}{\frac{n_{1}\times n_{2}}{n_{1}+n_{2}}\left( n_{1}+ n_{2}-4 \right)} \right)$.

Recent simulations by Hamman et al. (2018) show that this method reduces a weighting bias introduced by the more commonly used formula for the sampling variance of *d* (Koricheva et al. 2013, p. 63)*,* especially when sample sizes are small and heterogeneity among studies is high, as is commonly the case in ecology and evolution (Senior et al. 2016).

To quantify differences in variability between treatment levels and between the sexes, we calculated *lnCVR*, the natural log of the ratio of the coefficients of variation of two groups, and its associated variance (Nakagawa et al. 2015). We used *lnCVR_treatment_* to compare the variability of each pairwise combination of treatment levels, and *lnCVR_sex_* to compare the variability of males and females measured at each treatment level.

Effect sizes were excluded from analyses when the reported standard deviation for one or both group means was zero, and where the pooled sample size for both groups was <5. In analyses of *lnCVR*, we also excluded cases where mean trait expression was zero.

***Meta-analytic models in a Bayesian framework.*** We implemented Bayesian meta-analysis models in *R* (v.3.2.1; R Core Development Team) using the *MCMCglmm* package (Hadfield, 2010). We used a flat improper prior (the default in *MCMCglmm*) for fixed effects, and defined inverse-Wishart priors with *nu* = 0.002 and *V* = 1 for all random effects and residual covariances. For each model, we tested convergence between three independent MCMC chains of 510000 iterations (with burn in of 10000 iterations and a thinning interval of 1000 iterations) using Gelman and Rubin’s (1992) diagnostic, applying the criterion that values of ~1 indicate convergence. We assessed autocorrelation within MCMC chains (criterion: autocorrelation <0.1) and inspected trace plots to confirm that chains mixed well. After confirming chain convergence, we ran each model again using a single chain (1530000 iterations with 30000 iteration burn in and 1000 iteration thinning interval) to obtain posterior estimates of parameters.

***Heterogeneity.*** Following Nakagawa and Santos (2012) we calculated *I^2^* statistics for the models described above. Total *I^2^* describes the proportion of heterogeneity among effect sizes that is not due to sampling error, and can be partitioned into *I^2^* estimates for each variance component in a model, plus residual variation. *I^2^_species_* refers to heterogeneity due to differences between species, while *I^2^_phylogeny_* refers to heterogeneity due to phylogenetic relatedness.

***Publication bias & methodological moderators.*** We assessed publication bias through visual inspection of funnel plots (meta-analytic residuals plotted against the precision of each estimate, 1/SE), and by using Egger’s regression to test statistically for funnel plot asymmetry. We explored temporal trends in effect size by including the (centered and standardized) year of publication as a moderator variable in our meta-regression models, as effect sizes within a research topic are often found to decrease in magnitude over time (Koricheva et al. 2013).

**SUPPLEMENTARY RESULTS**

**Theoretical Predictions: cyclic changes in the environment**

When changes in the environment are cyclic, solutions to the long-run evolutionary dynamics of the system are considerably more complex and, as a result, more difficult to interpret. To simplify the presentation, we therefore focus on the case where additive genetic variances and strengths of stabilizing selection are symmetric between the sexes (*V_A_* = *V_A,f_* = *V_A,m_*; $\gamma=\gamma_{f}=\gamma_{m}$). Under female demographic dominance (from eq. (4)), the mean intrinsic growth rate at steady-state becomes:

|  | $\bar{r}=r_{\max}-\frac{1}{2\gamma}\mathrm{var}\left( \beta_{f} \right)$ | (13), |
| --- | --- | --- |

where $\mathrm{var}\left( \beta_{f} \right)$ is the variance across a steady-state cycle of the linear selection gradients for females (where the female selection gradient at a given time is $\beta_{f}=\partial\ln\left( \bar{W}_{f} \right)/\partial\bar{z}_{f}$). In contrast, with equal contributions of females and males to population dynamics (from eq. (5)), the mean intrinsic growth rate at steady-state is:

|  | $\bar{r}=r_{\max}-\frac{1}{4\gamma}\mathrm{var}\left( \beta_{f} \right)-\frac{1}{4\gamma}\mathrm{var}\left( \beta_{m} \right)$ | (14), |
| --- | --- | --- |

where $\mathrm{var}\left( \beta_{m} \right)$ is the variance of male selection gradients over a steady-state cycle ($\beta_{m}=\partial\ln\left( \bar{W}_{m} \right)/\partial\bar{z}_{m}$). The general solutions for $\mathrm{var}\left( \beta_{f} \right)$ and $\mathrm{var}\left( \beta_{m} \right)$ are:

| $\mathrm{var}\left( \beta_{f} \right)=2\left( A\gamma c^{2} \right)^{2}\left( \frac{2\left( B_{avg}-b_{avg} \right)}{\left( V_{A}\gamma\left( 1+r_{mf} \right) \right)^{2}+4c^{2}}-\frac{\left( B_{SD}-b_{SD} \right)}{\left( V_{A}\gamma\left( 1-r_{mf} \right) \right)^{2}+4c^{2}} \right)^{2}+\frac{\left( AV_{A}\gamma^{2}c \right)^{2}}{2}\left( \frac{2\left( B_{avg}-b_{avg} \right)\left( 1+r_{mf} \right)}{\left( V_{A}\gamma\left( 1+r_{mf} \right) \right)^{2}+4c^{2}}-\frac{\left( B_{SD}-b_{SD} \right)\left( 1-r_{mf} \right)}{\left( V_{A}\gamma\left( 1-r_{mf} \right) \right)^{2}+4c^{2}} \right)^{2}$ | (15a), |
| --- | --- |

and

| $\mathrm{var}\left( \beta_{m} \right)=2\left( A\gamma c^{2} \right)^{2}\left( \frac{2\left( B_{avg}-b_{avg} \right)}{\left( V_{A}\gamma\left( 1+r_{mf} \right) \right)^{2}+4c^{2}}+\frac{\left( B_{SD}-b_{SD} \right)}{\left( V_{A}\gamma\left( 1-r_{mf} \right) \right)^{2}+4c^{2}} \right)^{2}+\frac{\left( AV_{A}\gamma^{2}c \right)^{2}}{2}\left( \frac{2\left( B_{avg}-b_{avg} \right)\left( 1+r_{mf} \right)}{\left( V_{A}\gamma\left( 1+r_{mf} \right) \right)^{2}+4c^{2}}+\frac{\left( B_{SD}-b_{SD} \right)\left( 1-r_{mf} \right)}{\left( V_{A}\gamma\left( 1-r_{mf} \right) \right)^{2}+4c^{2}} \right)^{2}$ | (15b), |
| --- | --- |

where $B_{avg}=\frac{1}{2}\left( B_{f}+B_{m} \right)$, $B_{SD}=B_{m}-B_{f}$, $b_{avg}=\frac{1}{2}\left( b_{f}+b_{m} \right)$ and $b_{SD}=b_{m}-b_{f}$.

Eqs. (15a-15b) simplify greatly under two extremes of the cyclic change model. First, in the limit of slow cyclic change (*c* 🡪 0), results of the cyclic-change model converge to those of the directional change model. For example, with female demographic dominance, the mean intrinsic growth rate becomes:

|  | $\bar{r}=r_{\max}-\frac{1}{\gamma}\left( \frac{AcK_{f}}{V_{A}} \right)^{2}\left( \frac{1-\alpha r_{mf}}{1-r_{mf}^{2}} \right)^{2}+O\left( c^{4} \right)$ | (16), |
| --- | --- | --- |

When *c* is small, so that terms of *O*(*c*^4^) can be neglected, then $\bar{r}$ under cyclic change is proportional to the steady-state intrinsic growth under directional change (eqs. (8) and (16) are equivalent for $\eta=\sqrt{2}Ac$). Consequently, under slow environmental cycles, interactions between *r_mf_* and $\alpha$ are identical, in terms of population growth, to those described above for the directional change model.

In the limit of fast cycles in the environment (large *c*), long-run growth is roughly independent of the quantitative genetic parameters (*e.g.*, independent of *V_A_* and *r_mf_*) because evolution, in this case, is slow relative to the tempo of the environmental cycle. Instead, long-run patterns of population growth or decline are determined by the amplitude of environmental change (*A*), the effective rates of change of the female and male optima (*K_f_*, *K_m_*), and the sensitivity of sex-specific fitness to deviations from the optimum (*i.e.*, the strengths of stabilizing selection on each sex: *γ_f_* and *γ_m_*). With fast cycles, the mean intrinsic growth under female-demographic dominance is well approximated by:

|  | $\bar{r}=r_{\max}-\frac{\gamma}{4}\left( AK_{f} \right)^{2}$ | (17a). |
| --- | --- | --- |

With equal effects of female and male adaptation on population dynamics, the mean intrinsic growth becomes:

|  | $\bar{r}=r_{\max}-\frac{\gamma}{8}\left( AK_{f} \right)^{2}-\frac{\gamma}{8}\left( AK_{m} \right)^{2}$ | (17b). |
| --- | --- | --- |

In either case, plasticity that closely matches changes in the optimum (*i.e*., plasticity that causes *K_f_* and *K_m_* to be small) will promote positive population growth and persistence.

**Additional meta-analytic results**

***Heterogeneity in estimates of Hedges’ d and effects of moderator variables***

Heterogeneity of effect sizes within each trait category was generally extremely high (*I^2^_total_* = 95.79 – 99.98%; Table 2). In most categories, a large proportion of this heterogeneity was attributable to differences between studies, and differences between experiments within studies*.* Differences between species and differences due to phylogenetic relatedness explained minimal heterogeneity in all trait categories (Table 2).

The moderator variables ‘treatment applied’, ‘trait measured’, and ‘temperature stress’ had varying effects on estimates of Hedges’ *d* in the different data subsets. In the cold resistance, development time and survival classes, *d* varied significantly with the type of treatment applied (Fig. S3). There was a notable difference in the mean strength and direction of effect between studies applying an acute temperature treatment (which reduced cold resistance, for example) and those applying a hardening, acclimation, or rearing temperature treatment (which tended to increase cold resistance). This may reflect that treatments in the acute category did not involve pre-exposure to experimental temperature conditions. Additionally, in the survival and gene expression classes the mean effect size depended on the direction of the temperature manipulation (i.e. whether individuals were exposed to extreme cold or extreme heat) (Fig S4). Finally, in the cold resistance class, studies that measured cold resistance as CTmin found significantly larger estimates of *d*, on average, than other measures of cold resistance; furthermore, females showed significantly stronger plastic response than males in this trait (Fig. S4). However, we note that this category contains a relatively large number of effects from a small number of studies, indicating a possible effect of the particular experimental design, location, or study population in explaining these estimates.

***Heterogeneity in estimates of lnCVR and effects of moderator variables***

Heterogeneity was again high in the analyses of both *lnCVR_treatment_* and *lnCVR_sex_* (*lnCVR_treatment_*: *I^2^_total_* = 86.72 – 96.57%, *lnCVR_sex_*: *I^2^_total_* = 69.02 – 92.37%; Tables S2, S3). The included random variables explained little of this heterogeneity across most trait classes. In both analyses, differences due to phylogenetic relatedness and/or species identity explained substantial heterogeneity among effect sizes in the heat resistance data subset. Differences between experiments explained heterogeneity in estimates of *lnCVR_treatment_* for the gene expression and survival classes, and differences between studies explained heterogeneity in both *lnCVR_treatment_* and *lnCVR_sex_* for the size and survival classes.

The moderator variables ‘treatment applied’ and ‘trait measured’ did not significantly affect estimates of *lnCVR_treatment_* for any trait class (Fig. S5). In the gene expression and size classes *lnCVR_treatment_* differed significantly between levels of the moderator ‘temperature stress’ (Fig. S6). None of the included moderator variables significantly affected estimates of *lnCVR_sex_* in any trait class (Fig. S7, S8), except for gene expression, in which the analysis including the moderator ‘treatment applied’ indicated that hardening treatments led to greater trait variability in females than in males.

***Little indication of publication bias***

To assess potential sources of publication bias, we inspected funnel plots of model residuals against estimate precision (1/SE), and used Egger’s regression to formally test for funnel plot asymmetry. No significant asymmetry in Hedges’ *d* or *lnCVR_sex_* was detected in any of the trait classes (Table S5). Egger’s tests indicated significant asymmetry in the funnel plots of *lnCVR_treatment_* for the gene expression and survival classes. However, whether these asymmetries indicate publication bias is doubtful, as coefficients of variation were not the focus of the original studies, and are therefore unlikely to have influenced publication decisions. Funnel plot asymmetries can result from a range of sources including heterogeneity between studies or study taxa (Jennions et al. 2013), and for both the gene expression and survival trait classes we saw significant heterogeneity in *lnCVR_treatment_* explained by the temperature manipulation applied.

Year of publication and its interaction with sex had no effect on Hedges’ *d* or *lnCVR_treatment_* in most trait categories (Table S5). For cold resistance studies *d* increased over time, but the difference between the sexes did not change significantly over time. Similarly, *d* decreased over time for studies of size, but there was no effect of the year by sex interaction on *d*. For survival studies a negative year by sex interaction indicated that *d* increased over time for females but not males; however, overall *d* did not differ significantly from zero for either sex (Table 3). For gene expression studies a positive year by sex interaction reflected decreasing *lnCVR_treatment_* over time for females but not males, and for size studies *lnCVR_treatment_* increased over time for females but not males. Year of publication and its interaction with sex had no effect on *lnCVR_sex_* in any trait category (Table S5).

**REFERENCES**

Abramoff, M. D., P. J. Magalhaes, and R. S. Ram. (2004). Image processing with ImageJ. *Biophotonics Int.,* 11, 36-42.

Bell, G. (2017). Evolutionary rescue. Annu. Rev. Ecol. Evol. Syst. , 48, 605-627.

Chevin, L.-M., R. Lande, and G. M. Mace. (2010). Adaptation, plasticity, and extinction in a changing environment: towards a predictive theory. *PLoS Biol.,* 8, e1000357.

Connallon, T., and M. D. Hall. (2016). Genetic correlations and sex‐specific adaptation in changing environments. *Evolution,* 70,2186-2198.

Crow, J. F., and M. Kimura. (1970). *An introduction to population genetics theory.* Harper and Row, New York.

Gelman, A., and D. B. Rubin. (1992). Inference from iterative simulation using multiple sequences. *Statist. Sci,.* 7, 457-472.

Hadfield, J. (2010). MCMC methods for multi-response generalized linear mixed models: The MCMCglmm R package. *J. Stat. Softw.,* 33, 1-22.

Hamman, E.A., Pappalardo, P., Bence, J.R., Peacor, S.D. & Osenberg, C.W. (2018) Bias in meta-analyses using Hedges *d*. *Ecosphere*, 9, e02419.

Hadfield, J. (2010). MCMC methods for multi-response generalized linear mixed models: The MCMCglmm R package. *J. Stat. Soft.,* 33, 1-22.

Harts, A. M., L. E. Schwanz, and H. Kokko. (2014). Demography can favour female-advantageous alleles. *P. Roy. Soc. B-Biol. Sci.,* 281, 20140005.

Hedges, L.V. (1982). Estimation of effect size from a series of independent experiments. *Psychol. Bull.,* 92, 490-499.

Hinchcliff, C., S. A. Smith, J. F. Allman, J. G. Burleigh, R. Chaudhary, et al. (2015). Synthesis of phylogeny and taxonomy into a comprehensive tree of life. *Phil. Trans. R. Soc. B.*,13, 12764-12769.

Jennions, M. D., C. J. Lortie, and M. S. Rosenberg. (2013). Publication and related biases. *Handbook of Meta-analysis in Ecology and Evolution.* Princeton University Press. Princeton and Oxford.

Koricheva, J., M. D. Jennions, and J. Lau. (2013). Temporal trends in effect sizes: causes, detection, and implications. *Handbook of Meta-analysis in Ecology and Evolution.* Princeton University Press Princeton and Oxford.

Lande, R. (1980). Sexual dimorphism, sexual selection, and adaptation in polygenic characters. *Evolution,* 34:292-305.

Lande, R., and S. Shannon. (1996). The role of genetic variation in adaptation and population persistence in a changing environment. *Evolution*, 50: 434-437.

Lynch M, and R. Lande. (1993). Evolution and extinction in response to environmental change. In: Kareiva, P., J. Kingsolver, R. Huey, eds. *Biotic interactions and global change*. Sunderland, MA: Sinauer. pp 234–250.

Nakagawa, S. and E. S. A. Santos. (2012). Methodological issues and advances in biological meta-analysis. *Evol. Ecol.,* 26: 1253-1274.

R Core Team (2016) R: a language and environment for statistical computing. Vienna, Austria: R Foundation for Statistical Computing. See https://www.R-project.org/

Rankin, D. J. and H. Kokko. (2007). Do males matter? The role of males in population dynamics. *Oikos* 116, 335-348.

Rasband, W. S. (1997). *ImageJ.* US National Institutes of Health, Bethesda, MD.

Senior, A.M., Grueber, C.E., Kamiya, T., Lagisz, M., O'Dwyer, K., Santos, E.S.A. & Nakagawa, S. (2016) Heterogeneity in ecological and evolutionary meta-analyses: its magnitude and implications. *Ecology,* 97, 3293-3299.

Teder, T., and T. Tammaru. (2005). Sexual size dimorphism within species increases with body size in insects. *Oikos,* 108:321-334.
